# Isolated physical activity programs during pregnancy and postpartum: Meta-analysis of randomized trials with usual-care controls on depressive and anxiety symptoms

**DOI:** 10.1007/s00737-026-01752-9

**Published:** 2026-09-14

**Authors:** Daniela Aparecida de Faria, Victor Antonio Ferreira Freire, Dayanne Gabriela de Melo Marques, Paulo Henrique Nogueira da Fonseca, Kelly de Freitas Santos, Crystian Bitencourt Oliveira, Luiza Oliveira Perucci, Marina de Barros Pinheiro, Melina Barros-Pinheiro

**Affiliations:** 1 Federal University of São João del-Rei, São João del Rei, Brazil; 2 The University of Sydney, Sydney, Australia; 3 University of California, San Diego, San Diego, United States

**Keywords:** Perinatal depression, Antenatal, Postpartum, Physical activity, Exercise, Randomized controlled trials, Meta-analysis, Anxiety

## Abstract

**Objective:**

This study systematically evaluated the isolated effects of physical activity on depressive and anxiety symptoms during the perinatal period.

**Methods:**

This systematic review was registered in PROSPERO (CRD42022301284) and conducted in accordance with the Cochrane Handbook and PRISMA guidelines. Randomized controlled trials were included if they assessed the effects of standalone physical activity interventions on depressive or anxiety symptoms in pregnant or postpartum women. Eligible trials involved physical activity interventions of any type, frequency, intensity, or duration, provided that the control group received usual care or no intervention without additional active components. Prevention-oriented trials and symptomatic/treatment-oriented trials were analyzed separately according to baseline population profile.

**Results:**

Out of 10,938 records identified, 17 trials met the inclusion criteria. In prevention-oriented trials, physical activity was associated with lower depressive symptom scores during both the antenatal period (SMD: -0.52, 95% CI: -0.75 to -0.30; I² = 46%; seven trials; low-certainty evidence) and the postpartum period (SMD: -0.33, 95% CI: -0.57 to -0.09; I² = 86%; seven trials; very low-certainty evidence). For antenatal anxiety symptoms, physical activity did not show a clear effect compared with no intervention (SMD: -0.03, 95% CI: -0.31 to 0.25; I² = 0%; three trials; very low-certainty evidence). In a separate exploratory analysis of symptomatic/treatment-oriented trials, physical activity was associated with lower postpartum depressive symptom scores compared with no intervention (SMD: -0.38, 95% CI: -0.72 to -0.04; I² = 28%; three trials; very low-certainty evidence). Exploratory secondary analyses suggested favorable effects of structured physical activity and programs conducted three times per week for approximately 60 min on depressive symptoms, although the certainty of evidence was low or very low.

**Conclusions:**

Isolated physical activity interventions during the antenatal and postpartum periods may be associated with lower depressive symptom scores, particularly in prevention-oriented trials. A separate exploratory synthesis also suggested lower postpartum depressive symptom scores among symptomatic/treatment-oriented populations. However, the certainty of evidence was low to very low, and substantial clinical and methodological heterogeneity limits confidence in the magnitude and generalizability of these findings. Evidence for antenatal anxiety symptoms remains insufficient and inconclusive.

**Supplementary Information:**

The online version contains supplementary material available at https://doi.org/10.1007/s00737-026-01752-9.

## Highlights

- Physical activity was associated with lower antenatal and postpartum depressive symptom scores in prevention-oriented trials.
- An exploratory synthesis also suggested lower postpartum depressive symptoms in symptomatic/treatment-oriented trials.
- Evidence for antenatal anxiety symptoms was insufficient and inconclusive.
- Structured programs and sessions performed three times per week for about 60 minutes may be associated with greater benefit, but certainty of evidence was low to very low.

**Supplementary Information:**

The online version contains supplementary material available at https://doi.org/10.1007/s00737-026-01752-9.

## Background

Depressive and anxiety symptoms are frequently reported by women during the perinatal period, which encompasses conception, pregnancy, and the postpartum period (Howard and Khalifeh 2020). Perinatal depression is defined as a depressive episode of varying severity occurring during pregnancy or within the first postpartum year (World Health Organization 2010, 2022a, 2022b). Previous studies have reported that the prevalence of depressive symptoms during pregnancy ranges from 10% to 40%, depending on the population, assessment instrument, timing of evaluation, and diagnostic threshold used (American College of Obstetricians and Gynecologists 2018; Gelaye et al. 2016; World Health Organization 2022a, 2022b). This high burden supports the need for preventive strategies that can be implemented safely during pregnancy and the postpartum period.

Perinatal anxiety refers to intense and persistent anxiety during the perinatal period, which differs from common anxiety due to its symptoms, significantly affecting the daily life and functioning of pregnant women (Al-Sabah et al. 2024). These symptoms include excessive worries about the baby’s health, fear of childbirth, anxiety about motherhood, panic attacks, suicidal thoughts, insomnia, and irritability. The global prevalence of perinatal anxiety is estimated to range from 15% to 23%, highlighting its significance as a public health issue (O’Hara and Wisner 2014). Together with depressive symptoms, anxiety symptoms represent an important target for early identification and prevention because they may coexist with depression and contribute to adverse maternal and infant outcomes.

Depression and anxiety during pregnancy and the postpartum period can have both immediate and long-term adverse effects on mothers and their children. For mothers, these conditions strongly predict postpartum depression and anxiety and are associated with an increased risk of suicide (Chin et al. 2022), reduced self-care, preterm labor, low birth weight, and difficulties in caregiving and bonding with the infant (Field 2010; Sandre et al. 2022; Fallon et al. 2016; Shrestha et al. 2024). Furthermore, maternal depression and anxiety have been linked to delays in the cognitive, emotional, and social development of offspring (Halligan et al. 2004; Girchenko et al. 2024; Bowman et al. 2022; Aoyagi and Tsuchiya 2019).

Treatment for perinatal depression and anxiety often includes antidepressants, anxiolytics, psychotherapy, and other non-pharmacological approaches (Fitelson et al. 2011). However, access, adherence, acceptability, safety concerns, and variable effectiveness may limit the reach of treatment-based approaches, particularly when symptoms are mild or subclinical (Kołomańska-Bogucka and Mazur-Bialy 2019). Concerns have also been raised about pharmacological treatments, including potential metabolic changes in the mother and drug exposure to the newborn via the placental barrier or breastfeeding (Fitelson et al. 2011). Therefore, safe and scalable preventive strategies are needed. As a non-pharmacological alternative, physical activity interventions have been proposed to reduce symptoms of depression and anxiety (Fitelson et al. 2011; Al-Sabah et al. 2024; Laurenzi et al. 2020).

Physical activity is recommended during pregnancy for most women without contraindications and may offer mental health benefits through biological, psychological, and behavioral pathways, including improved sleep, reduced perceived stress, enhanced self-efficacy, social interaction, and modulation of inflammatory and neuroendocrine responses (Mottola et al. 2018; Fitelson et al. 2011; Laurenzi et al. 2020). Previous systematic reviews and meta-analyses have suggested that physical activity or exercise may reduce depressive symptoms during pregnancy and the postpartum period (Poyatos-León et al. 2015; Davenport et al. 2018; Kołomańska-Bogucka and Mazur-Bialy 2019; Liu et al. 2023). However, the evidence remains heterogeneous, and some syntheses have included multicomponent interventions, active control groups, mixed antenatal and postpartum outcomes, or populations with different baseline symptom profiles, which may limit the interpretation of physical activity as an isolated preventive strategy.

Although physical activity has been associated with better perinatal mental health, prior reviews often combined antenatal and postpartum data, included multicomponent interventions or active controls, and did not consistently account for baseline symptom severity or implementation features (Davenport et al. 2018; Carter et al. 2019; He et al. 2023; Liu et al. 2022; Ji et al. 2024; Shuai et al. 2024). We therefore synthesized only randomized trials of isolated physical activity programs versus usual care without additional intervention, separating antenatal and postpartum outcomes and describing modality, supervision, frequency/intensity, adherence, and, when available, the timing of initiation during pregnancy. This design allows a more specific assessment of the independent association between physical activity and depressive or anxiety symptoms during the perinatal period.

## Methods

This systematic review was prospectively registered in PROSPERO (CRD42022301284) and conducted in accordance with the Cochrane Handbook for Systematic Reviews of Interventions (Higgins et al. 2022) and the Preferred Reporting Items for Systematic Reviews and Meta-Analyses (PRISMA) guidelines (Page et al. 2021).

### Eligibility criteria

#### Study design

This review included only randomized controlled trials (RCTs) evaluating standalone physical activity interventions compared with usual care without additional intervention.

#### Population

Eligible studies enrolled pregnant or postpartum women in the perinatal period. No restriction was applied regarding participants’ age, parity, gestational age at enrollment, mode of delivery, or baseline obstetric risk, provided that the study met all other eligibility criteria and the physical activity intervention was not combined with another active component. Trials were classified according to baseline population profile. Prevention-oriented trials were defined as those in which women were not recruited on the basis of depression/anxiety diagnosis, elevated depressive or anxiety symptoms, at-risk status, or active treatment at baseline. Trials enrolling women with depression/anxiety diagnosis, elevated symptoms, at-risk status, or active treatment at baseline were classified as symptomatic/treatment-oriented trials and analyzed separately.

#### Intervention

Studies investigating any form of physical activity were eligible, regardless of the type, frequency, intensity, duration, or modality of the intervention. Physical activity and exercise were defined according to the World Health Organization (WHO) guidelines (Caspersen et al. 1985; World Health Organization 2020). Physical activity refers to any bodily movement produced by skeletal muscles that requires energy expenditure, while exercise is a subcategory of physical activity that is planned, structured, repetitive, and aimed at improving or maintaining one or more components of physical fitness (Dasso 2019). Both physical activities integrated into daily routines and structured exercise programs were included, irrespective of whether the studies distinguished between the two terms.

To ensure that the effects of physical activity were evaluated in isolation, only studies where physical activity was applied as a standalone intervention were included. Studies that combined physical activity with other interventions (e.g., psychosocial, pharmacological, or dietary programs) were excluded to avoid confounding effects, allowing for the independent impact of physical activity on the outcomes of interest to be assessed.

#### Comparison

Only studies comparing physical activity interventions with comparators receiving usual care - without any additional interventions or structured physical activity prescription/counseling beyond routine care - were included.

#### Outcomes

The primary outcomes of this review were depressive and anxiety symptom scores during the perinatal period, assessed separately for the antenatal and postpartum periods. Studies were included if these symptoms were assessed using validated self-reported measures, such as the Edinburgh Postnatal Depression Scale (EPDS), Center for Epidemiological Studies Depression Scale (CES-D), State Anxiety Inventory (S-AI), State-Trait Anxiety Inventory (STAI), Profile of Mood States (POMS), Inventory of Depression and Anxiety Symptoms (IDAS), or Hamilton Depression Rating Scale (HDRS), or through diagnosis based on expert interviews using International Classification of Diseases (ICD) codes.

Secondary and exploratory analyses focused on intervention characteristics and implementation features, including type or modality of physical activity, frequency per week, session duration, intensity, program duration, professional supervision, adherence, and timing of initiation during pregnancy when available. These analyses were conducted to describe the interventions and explore whether specific characteristics of physical activity programs were associated with different effects on depressive or anxiety symptoms.

### Search strategy

Searches were conducted in the following electronic databases: MEDLINE via PubMed, EMBASE, PEDro, PsycInfo, and SPORTDiscus. Detailed search strategies are available in Supplement 1. No restrictions were applied regarding language or date of publication. The initial search was performed in Febr

uary 2022 and updated in February 2024, and subsequently updated on 14 June 2026 to cover records published or indexed from February 2024 to June 2026. The same conceptual search blocks and eligibility criteria were maintained, with database-specific syntax and descriptors checked and adapted when necessary.

### Study selection

Two independent reviewers (DGMM and PHNF) screened titles and abstracts and assessed full texts using COVIDENCE software. Disagreements were resolved through discussion, and when necessary, a third reviewer (DAF) was consulted. The degree of agreement between the two reviewers was evaluated using the Kappa Coefficient, with a 95% confidence interval.

### Data extraction

The extracted data included: author, year, country, study design, number of participants at baseline and follow-up for intervention and control groups, characteristics of the intervention (e.g., frequency, intensity, type of exercise, professional supervision, duration), and outcomes (e.g., depression and anxiety levels, depressive symptoms). Mean estimates were extracted hierarchically as follows: mean difference, change score, and final score. Any discrepancies were resolved by consensus. Corresponding authors of included studies were contacted to obtain missing information when necessary.

### Risk of bias

The risk of bias was assessed using the PEDro scale, a validated tool for evaluating the methodological quality of randomized controlled trials (Maher et al. 2003). The PEDro scale includes 11 items addressing aspects such as random allocation, concealed allocation, baseline comparability, blinding, follow-up adequacy, and appropriate statistical analysis. Each item is scored as “yes” (1 point) or “no” (0 points), with the final score ranging from 0 to 10, as the first item (external validity) is not included in the total score. Higher scores indicate better methodological quality and lower risk of bias, ensuring consistent and standardized evaluations across studies.

Two reviewers independently assessed methodological quality using the PEDro scale. Disagreements were resolved by discussion, and when consensus could not be reached, a third reviewer was consulted. Interrater agreement was interpreted according to McHugh (2012), with kappa values between 0.61 and 0.80 considered substantial agreement.

### Assessment of level of evidence

The certainty of evidence was assessed with the GRADE approach (Schünemann et al. 2013a, 2013b; Guyatt et al. 2011a, 2011b; Deeks et al. 2024) (see Supplements 2–3). For inconsistency, we interpreted heterogeneity contextually, avoiding rigid I² thresholds, considering the direction and magnitude of effects, overlap of confidence intervals, and clinical/methodological diversity across trials (Schünemann et al. 2013a, 2013b; Guyatt et al. 2011a, 2011b; Deeks et al. 2024; Schünemann et al. 2024). When inconsistency was substantial by this integrated appraisal, we downgraded the certainty. For imprecision, we downgraded when the total information size was low and/or the confidence intervals included both clinically unimportant and important effects (Guyatt et al. 2011a, 2011b).

### Data synthesis

Meta-analyses were conducted using random-effects models to compute mean differences (MDs) with 95% confidence intervals (CIs) when outcomes were measured using the same instrument. For comparisons involving different instruments, standardized mean differences (SMDs; Hedges g) with 95% CIs were computed. We required a minimum of three studies per outcome for quantitative synthesis. Given the expected clinical and methodological heterogeneity across trials, the systematic review was considered the primary framework for evidence synthesis, while meta-analyses were conducted as exploratory quantitative summaries when studies were sufficiently comparable in terms of population, intervention, comparator, and outcome.

Primary quantitative syntheses focused on prevention-oriented trials, defined as trials in which women were not recruited on the basis of depression/anxiety diagnosis, elevated depressive or anxiety symptoms, at-risk status, or active treatment at baseline. Trials enrolling women with depression/anxiety diagnosis, elevated symptoms, at-risk status, or active treatment at baseline were classified as symptomatic/treatment-oriented trials and were analyzed separately from prevention-oriented trials. When at least three studies were available for the same outcome, these trials were pooled in a separate exploratory meta-analysis; otherwise, they were summarized narratively.

Comparisons were stratified by baseline population profile, antenatal versus postpartum period, and, when data allowed, intervention characteristics. Secondary and exploratory meta-analyses were conducted according to intervention characteristics when at least three studies were available for pooling. These analyses included type or modality of physical activity, frequency/session duration, and intensity. Exercises were categorized into two groups—structured exercise and recreational activity—following the definitions provided by Baldwin, Hassett, and Sherrington (Baldwin et al. 2023). When quantitative pooling was not possible, implementation characteristics such as supervision, adherence, program duration, and timing of initiation were summarized descriptively.

Heterogeneity was quantified using I² and interpreted contextually, avoiding rigid thresholds and considering the magnitude and direction of effects, overlap of confidence intervals, and clinical/methodological diversity across trials. When a mean difference was applied, we explicitly reported the underlying instrument scale (e.g., EPDS 0–30). When multiple instruments were combined, we used standardized mean differences. For each outcome, the final follow-up assessment was used to evaluate the sustainability of intervention effects over the long term. When studies reported data as medians and ranges, means and standard deviations were estimated using the method described by Wan et al. (2014).

Given the expected clinical and methodological heterogeneity across physical activity interventions, random-effects models were used. For meta-analyses including at least five studies, 95% prediction intervals were calculated to describe the expected range of effects in future comparable settings. Sensitivity analyses were conducted by excluding trials with lower methodological quality, defined as PEDro scores < 6. Heterogeneity was explored through prediction intervals, subgroup analyses based on intervention characteristics, and sensitivity analyses based on methodological quality. Subgroup analyses were planned to explore potential sources of heterogeneity and were aligned with the review protocol, which included secondary questions regarding intervention characteristics such as type of exercise, duration, dose, progression, intensity, and level of supervision. Given the limited number of trials within several subgroups and the substantial between-study heterogeneity, these analyses were interpreted as exploratory and hypothesis-generating. Meta-regression was not performed because the number of studies per covariate was insufficient to support reliable model estimation. All statistical analyses were conducted using RevMan version 5.4 software (Cochrane, UK). To contextualize our scope against prior syntheses, a side-by-side comparison is provided in Supplementary Table S4.

## Results

**Fig. 1:**
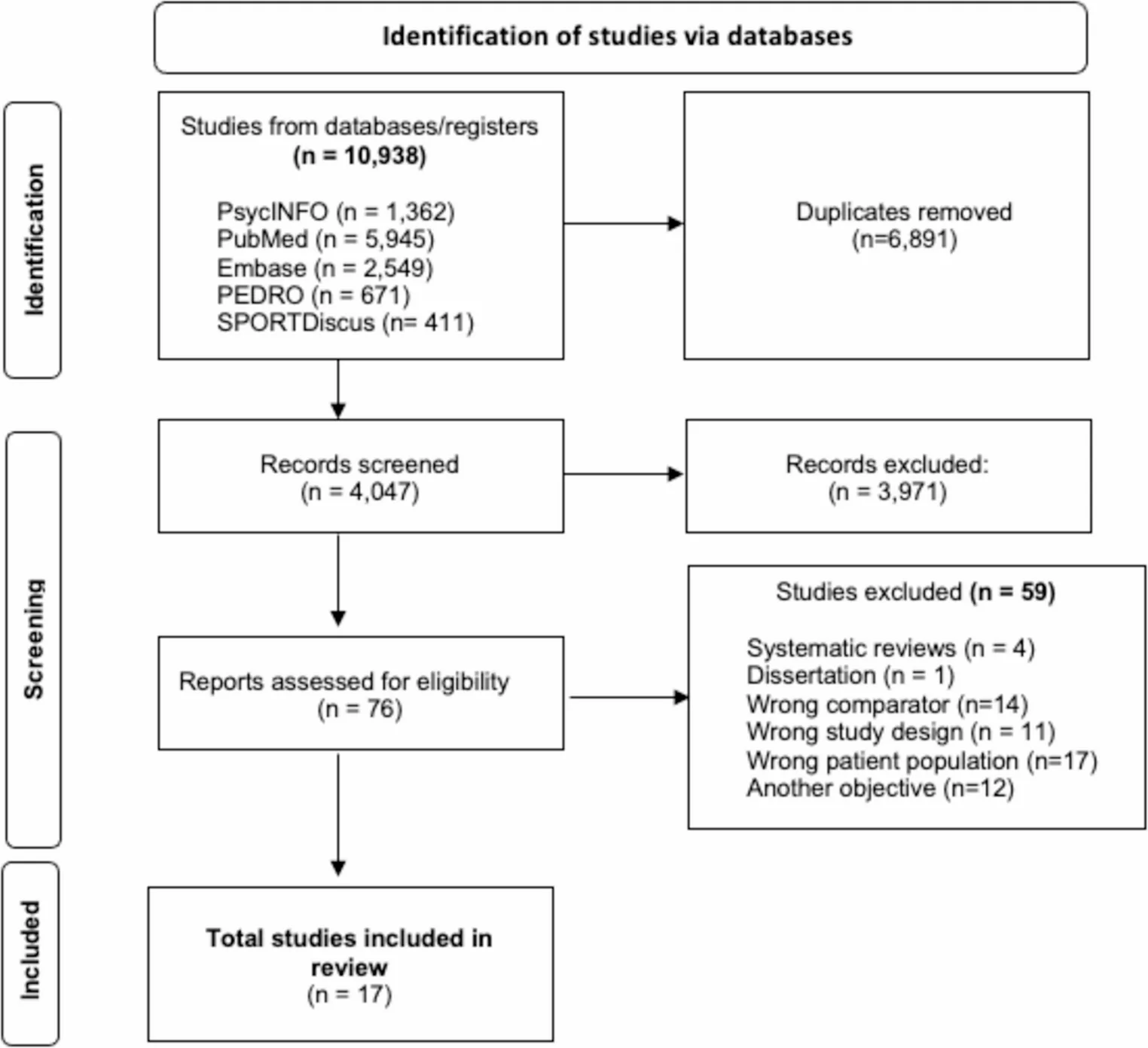
Flowchart of the selected studies included in this review

After the updated search conducted in June 2026, a total of 10,938 records were identified through database searches. After removal of 6,891 duplicates, 4,047 records were screened by title and abstract. Of these, 3,971 records were excluded, and 76 reports were assessed for eligibility in full text. Fifty-nine reports were excluded after full-text assessment because they were systematic reviews (*n* = 4), dissertation (*n* = 1), had a wrong comparator (*n* = 14), wrong study design (*n* = 11), wrong patient population (*n* = 17), or intervention/outcome not aligned with the review question (*n* = 12). Ultimately, 17 randomized controlled trials met the inclusion criteria and were included in this systematic review. The review process is outlined in Fig. 1. The inter-rater reliability between the two researchers was substantial, with a kappa coefficient of 0.63, indicating substantial agreement (McHugh 2012).

Table 1 describes the characteristics of the 17 included randomized controlled trials. Fourteen trials were classified as prevention-oriented trials, in which women were not recruited on the basis of depression/anxiety diagnosis, elevated symptoms, at-risk status, or active treatment at baseline. Three trials were classified as symptomatic/treatment-oriented trials because women were recruited with elevated symptoms, at-risk status, diagnosis, or active treatment at baseline. Prevention-oriented and symptomatic/treatment-oriented trials were not combined in the same quantitative synthesis. Five trials were conducted in Spain (Perales et al. 2015a, 2015b; Perales et al. 2016; Aguilar-Cordero et al. 2019; Navas et al. 2021), two in the United States (Buttner et al. 2015; Avery et al. 2026), and the remaining studies were conducted in Taiwan (Heh et al. 2008), Colombia (Robledo-Colonia et al. 2012), Iran (Mohammadi et al. 2015), Brazil (Coll et al. 2019), China (Rong et al. 2021), Japan (Taniguchi and Sato 2016), Australia (Norman et al. 2010), Norway (Songøygard et al. 2012), the United Kingdom (Daley et al. 2015), and Italy (Saccone et al. 2026). The total randomized sample sizes ranged from 20 to 855 participants, with age data reported as means, standard deviations, or ranges across studies.

**Table 1:** Characteristics of randomized controlled trials evaluating the impact of physical activity on depressive and anxiety symptoms during the perinatal period compared to no intervention

Author, Year, Country/ PEDro score	Participants Allocated/Analyzed [*n*, age (mean (SD)]	Study objectives Guidelines, assessment instrument, and cutoff/eligibility criterion	Intervention details/type of physical activity	Outcome and results	Follow up (weeks)	Results (mean ± SD) *n*
Prevention-oriented trials: women not recruited on the basis of depression/anxiety diagnosis, elevated symptoms, or active treatment						
Robledo-Colonia et al. 2012; Colombia/7	**A. Intervention** n: 80(randomized); n: 40/37 (allocated/analyzed) Age: 16 to 30 y/o 21 (3) y/o **B. Control** No intervention n: 40/37 (randomized); (allocated/analyzed) Age: 16 to 30 y/o 21 (3) y/o	To evaluate whether 3-month supervised aerobic exercise decrease depressive symptoms in nulliparous women.	Frequency: 3 times a week/3 months	Antenatal depression A supervised 3-month program reduced their depressive symptoms on the CES-D questionnaire by 4 points (95% CI 1 to 7) more than the control group.	**A.** Intervention 3 months	A. 10 (6) *n* = 37
		Guidelines: ACSM and ACOG Assessment instrument: CES-D Cutoff/eligibility criterion: CES-D ≥ 16 reported as the cut-point for depression; not used as an eligibility criterion. Women with psychiatric diseases were excluded. Classification: prevention-oriented trial.	Intensity: moderate-vigorous		**B.** Control 3 months	**B.** 16 (8) *n* = 37
			Physical activity modality/classification: pram-walking/structured exercise;			
			Professional assistance: physical therapist Duration: 60 min			
Mohammadi et al. 2015; Iran/7	**A. Intervention** n: 127 (randomized); n: 43/38 (allocated/analyzed) Age: 25.2 (4.7) y/o **B. Control** Usual care *n* = 42/(35–36) (randomized); (allocated/analyzed) 25.3 (5.20) y/o	To determine the effectiveness of home-based antenatal and postnatal on depression and fatigue in the 1 and 2 months postpartum.	Frequency: 3 times a week/until delivery	Postpartum depression This study did not provide evidence to show that home-based exercises during pregnancy or during pregnancy and post-partum period have a preventive effect on post-partum depression and fatigue.	**A.** Intervention Week-4 PPD Week-8 PPD	**A.** 7.66 (5.46) 6.58 (4.63) *n* = 38
			Intensity: low-intensity			
		Guideline: not specified Assessment instrument: EPDS Cutoff/eligibility criterion: EPDS < 15 required at baseline; women currently suffering from depression or with other known psychiatric disorders were excluded. EPDS ≥ 13 was reported for outcome classification, not eligibility. Classification: prevention-oriented trial.	Physical activity modality/classification: mixed physical activity: low-intensity stretching and breathing exercises/structured exercise;		**B.** Control Week-4 PPD Week-8 PPD	**B.** 7.46 (4.5); *n* = 35 6.5 (5.12); *n* = 36
			Professional assistance: Not specified			
			Duration: 20–30 min			
Perales et al. 2015a, Spain/6	**A. Intervention** n:187(randomized); n: 101/90 (allocated/analyzed) Age: 31.08 (3.36) y/o **B. Control** General care n: 83/77 (randomized); (allocated/analyzed) Age: 31.66 (3.86) y/o	To assess whether a supervised exercise and specific physical exercise program can reduce depressive symptoms in pregnant women.	Frequency: 3 times a week/(28–30 weeks)	Antenatal depression Supervised physical exercise during pregnancy reduces the level of depression and its incidence in pregnant women.	**A.** Intervention 1 st trimester 9–12 weeks 3rd trimester 38–39 weeks **B.** Control 1 st trimester 9–12 weeks 3rd trimester 38–39 weeks	**A**. 9.87(8.9) 7.67 (6.3) *n* = 90 **B.** 9.38 (8.1) 11.34 (9.74) *n* = 77
			Intensity: light-moderate			
		Guideline: ACOG Assessment instrument: CES-D Cutoff/eligibility criterion: CES-D ≥ 16 was considered indicative of depression; not used as an eligibility criterion. Depression was assessed at baseline and end pregnancy, and some participants had CES-D ≥ 16 at baseline. Classification: prevention-oriented trial based on recruitment criteria; not selected for depressive symptoms, although not strictly asymptomatic at baseline.	Physical activity modality/classification: mixed: Walking, static stretching, pelvic floor muscle training/structured exercise; Professional assistance: qualified fitness specialist with the assistance of an obstetrician. Duration: 60 min			
Aguilar-Cordero et al. 2019; Spain/6	**A. Intervention** n:140 (randomized); n: 70/65 (allocated/analyzed) Age: 21 to 43 y/o 34.52 **(**4.50) y/o **B. Control** Usual care n: 70/64 (randomized); (allocated/analyzed) Age: 21 to 43 y/o 33.67 **(**5.37) y/o	To determine whether physical activity during pregnancy alleviates PPD Guideline: ACOG Assessment instrument: EPDS Cutoff/eligibility criterion: EPDS ≥ 10 used to indicate risk of postpartum depression and EPDS > 16 to indicate more severe risk; not used as an eligibility criterion.	Frequency: 3 times a week/17-week	Postpartum depression Women who did moderate physical exercises in an aquatic environment are at lower risk of PPD than to sedentary women.	**A**. Intervention After delivery	**A.** 6.41 (3.68) *n* = 65
			Intensity: moderate			
			Physical activity modality/classification: physical activity mixed (in an aquatic environment: heating; the main phase, in which the activity is divided into an aerobic session followed by strength and resistance exercises; and final stretching and relaxation/structured exercise; Professional assistance: not specified Duration: 60 min		**B.** Control After delivery	**B.** 10.17 (2.38) *n* = 64
		Classification: prevention-oriented trial.				
Coll et al. 2019; Brazil/5	**A. Intervention** n: 639 (randomized); n: 213/192 (allocated/analyzed) Age: >18 y/o 27.20 (5.50) y/o	To assess the efficacy of a 16-week regular exercise during pregnancy on the prevention PPD Guideline: ACOG Assessment instrument: EPDS Cutoff/eligibility criterion: EPDS ≥ 12 used as positive screening for postpartum depression; not used as an eligibility criterion. Baseline depressive symptoms were not assessed. Classification: prevention-oriented trial.	Frequency: 3 times a week/16-week	Postpartum depression There was no significant difference in mean (SD) scores for postpartum depression between the intervention group and the control group.	**A.** Intervention Week-12 PPD	**A.** 4.8 (3.7) *n* = 192
			Intensity: moderate		**B.** Control Week-12 PPD	**B.** 5.4 (4.1) *n* = 387
	**B. Control** No intervention (daily activities) n: 426/387 (randomized); (allocated/analyzed) Age: >18 y/o 27.30 (5.50) y/o		Physical activity modality/classification: mixed: aerobic activities, strength training, and floor exercises/structured exercise;			
			Professional assistance: instructors with at least 1 year of postgraduate experience			
			Duration: 60 min			
Navas et al. 2021; Spain/7	**A. Intervention** n: 294 (randomized); n: 148/145 (allocated/analyzed) Age: 18 to 40 y/o 31.1 (4.1) **B. Control** No intervention (Standard antenatal care) n: 146/141 (randomized); (allocated/analyzed) Age: 18 to 40 y/o 31.5 (4.2) y/o	To analyze the effectiveness and safety of a moderate aerobic water exercise program on risk of PPD, sleep problems, and quality of life in women at one month after delivery Guideline: ACSM Assessment instrument: EPDS Cutoff/eligibility criterion: EPDS > 10 used to indicate risk of depression at 1 month postpartum; not used as an eligibility criterion. Severe psychiatric disorder was excluded. Classification: prevention-oriented trial.	Frequency: 3 times a week/5 months	Postpartum depression Women in the intervention group were less likely to report depression and had a lower mean EPDS score compared to control.	**A.** Intervention Week-4 PPD	**A.** 6.1 (1.9) *n* = 139
			Intensity: moderate		**B.** Control Week-4 PPD	**B.** 6.8 (2.4) *n* = 132
			Physical activity modality/classification: aerobic water exercise/structured exercise;			
			Professional assistance: not specified.			
			Duration: 45 min			
Saccone et al. 2026; Italy/7	**A. Intervention** n: 398 (randomized); n:199 (analyzed); Age: 33.9 (3.2) y/o. **B. Control** n: 398 (randomized); n: 199 (analyzed); Age: 33.8 (3.1) y/o.	To evaluate whether regular aerobic exercise during pregnancy reduces the incidence of postpartum depression in women with low-risk singleton pregnancies. Guideline: ACOG Assessment instrument: EPDS; SCID-5/DSM-V among women with EPDS ≥ 9 Cutoff/eligibility criterion: EPDS ≥ 12 used as primary outcome; EPDS ≥ 9 used as secondary outcome and for SCID-5/DSM-V assessment. Not used for eligibility. Prior PPD and prior/current psychiatric disorders were excluded. Classification: prevention-oriented trial.	Frequency: 3 times/week until 35 weeks’ gestation. Intensity: aerobic exercise; intensity not fully specified in table, but programme based on ACOG standards. Physical activity modality/classification: structured aerobic exercise including gradual warm-up, aerobic/resistance exercise, pelvic floor strengthening, coordination and balance exercises, stretching, and relaxation. Professional assistance: structured/supervised programme. Duration: 60 min.	Postpartum depression Women in the exercise group had lower rates of EPDS ≥ 12 and EPDS ≥ 9 at 3 months postpartum, and lower mean EPDS scores compared with controls.	**A**. Intervention 3 months postpartum **B**. Control 3 months postpartum	**1 A**. 5.1 (3.7), *n* = 199 EPDS ≥ 12: 12/199; EPDS ≥ 9: 35/199 2 A. DSM-V PPD: 13/199 1B. 7.1 (5.2), *n* = 199 EPDS ≥ 12: 40/199 EPDS ≥ 9: 63/199 **2B**. DSM-V PPD: 22/199
Rong et al. 2021; China/8	**A. Intervention** n: 64 (randomized); n: 32 (allocated/analyzed) Age: >18 y/o 29 (2.81) y/o **B. Control** No intervention (prenatal care) n: 32 (randomized); (allocated/analyzed) Age: >18 y/o 28.16 (2.78) y/o	To evaluate the efficacy of yoga on physiological and psychological discomforts in the pregnancy as well as on prenatal depression, prenatal anxiety, childbirth self-efficacy, and delivery outcomes in Chinese primiparas Guideline: Canadian Guideline for Physical Activity throughout Pregnancy Assessment instrument: EPDS **(1)** for prenatal depressive symptoms; S-AI/STAI-State **(2)** for prenatal anxiety symptoms Cutoff/eligibility criterion: No cutoff used as an eligibility criterion. No cutoff for depression or anxiety classification was reported; EPDS and S-AI/STAI-State scores were analyzed as continuous outcomes. Classification: prevention-oriented trial.	Frequency: 3 times a week/12-week	Antenatal depression and anxiety There was no difference in scores of depression and anxiety after the 12-week yoga exercise between the intervention and control.	**A**. Intervention After 12-week intervention	**1 A.** 7.16 (3.16) *n* = 32 **2 A.** 34.41 (6.97) *n* = 32
			Intensity: not specified			
			Physical activity modality/classification: Yoga/recreation			
			Professional assistance: yoga instructor.			
			Duration: 60 min		**B.** Control After 12-week intervention	**1 B.** 8.03 (4.08) *n* = 32 **2 B.** 34.31 (8.97) *n* = 32
Avery et al. 2026; USA/7	**A. Intervention** n: 20/10 randomized/analyzed; Age: 28.2 (4.6) y/o. **B. Control** n: 20/10 randomized/analyzed; Age: 30.4 (3.5) y/o.	To determine the impact of a 6-week prenatal yoga intervention on hip mobility, mental health, and delivery outcomes in pregnant women. Guideline: not specified Assessment instrument: EPDS **(1)** for depressive symptoms; STAI **(2)** for anxiety symptoms Cutoff/eligibility criterion: No cutoff used as an eligibility criterion. No cutoff for depression or anxiety classification was reported; EPDS and STAI scores were analyzed as continuous outcomes. Classification: prevention-oriented trial.	Frequency: at least 1 class/week for 6 weeks; classes were offered twice weekly. Intensity: not specified. Physical activity modality/classification: prenatal yoga/recreation. Each 60-min class included warm-up, yoga poses, balance poses, static stretches, dynamic movements adapted to pregnancy, and guided relaxation. Professional assistance: registered prenatal yoga instructor. Duration: 60 min.	Antenatal depression and anxiety Anxiety and depression improved numerically in the yoga group, but between-group differences in change scores were not statistically significant. The study was exploratory and underpowered.	**A**. Intervention Baseline After 6-week intervention **B**. Control Baseline After 6-week intervention	**1 A**. 8.9 (5.2) **2 A**. 43.5 (13.1) *n* = 10 **1 A**. 6.1 (5.7) **2 A**. 36.8 (7.0) *n* = 10 **1B**. 6.4 (5.6) **2B**. 37.2 (8.2) *n* = 10 **1B**. 6.9 (3.7) **2B**. 37.9 (11.5) *n* = 10
Perales et al. 2016; Spain/7	A. **Intervention** n: 241 (randomized); n: 120/83 (allocated/analyzed) Age: 31 (4) y/o B. **Control** Standard care n: 121/59 (randomized); (allocated/analyzed) Age: 31 (4) y/o	To assess the effects of an exercise program performed over pregnancy (from gestational weeks 9–11 to weeks 38–39) on the echocardiographic indicators and maternal cardiovascular disease risk factors. Guideline: ACOG Assessment instrument: CES-D Cutoff/eligibility criterion: CES-D ≥ 16 was considered indicative of depression; not used as an eligibility criterion. Depression was assessed at baseline and end pregnancy, and some participants had CES-D ≥ 16 at baseline. Classification: prevention-oriented trial based on recruitment criteria; not selected for depressive symptoms, although not strictly asymptomatic at baseline.	Frequency: 3 times per week (28–29 weeks)	Antenatal depression There is tendency toward a lower prevalence of depression at end pregnancy in the exercise group compared with controls.	**A.** Intervention Start (weeks 9–11) End of pregnancy (weeks 38–39) **B**. Control Start (weeks 9–11) End of pregnancy (weeks 38–39)	**A.** 8 (0–34)^a^; 7 (0–23)^a^ *n* = 83 **B.** 9 (0–41)^a^; 10 (0–41)^a^ *n* = 59
			Intensity: light–moderate			
			Physical activity modality/classification: walking (aerobic), stretching and pelvic floor exercises, and cool down period including relaxing exercises (visualization techniques and progressive muscle relaxation), static stretching, and partner massages/structured exercise;			
			Professional assistance: fitness instructor			
			Duration: 55–60 min			
Taniguchi and Sato 2016, Japan/7	A. **intervention** n: 118 (randomized); n: 60/54 (allocated/analyzed) Age: 22–36 y/o 28.5 (3.5) y/o **B. Control** Normal daily lives n: 58/53 (randomized); (allocated/analyzed) Age: >18 y/o 29.5 (3) y/o	To examine the effects of home-based walking on pregnancy outcomes and mood among sedentary women. Guideline: ACOG; Japanese Society of Clinical Sports Medicine Assessment instrument: POMS, including depression/depression–dejection **(1)** and tension–anxiety subscales **(2)** Cutoff/eligibility criterion: No cutoff used as an eligibility criterion. No cutoff for depression or anxiety classification was reported; POMS subscale scores were analyzed as mood-state outcomes. Women with self-reported physical, mental, or social problems or psychiatric drug use were excluded. Classification: prevention-oriented trial.	Frequency: 3 or more times per week/30- week	Antenatal depression and anxiety POMS scores at 38weeks’ pregnancy were lower than at pre-intervention, reveled that the unsupervised walking to reduce depression among previously sedentary pregnant women. There is not difference for anxiety outcome.	**A.** Intervention Baseline 38 weeks´	**1 A.** 5.5 (4.5)^b^ 3.0 (4.5)^b^ *n* = 54 **2 A.** 8 (7)^b^ 8 (8)^b^ *n* = 54
			Intensity: not specified		**B.** Control Baseline 38 weeks´	**1B.** 3.0 (8) 1.0 (7.5) *n* = 53 **2B** 8 (7)b 8 (11)b *n* = 53
			Physical activity modality/classification: home-base walking/structured exercise;			
			Professional assistance: unsupervised			
			Duration: 30 min			
Norman et al. 2010; Australia/7	**A.** I**ntervention** n: 161 (randomized); n: 80/62 (allocated/analyzed) Age: 29.3 (4) y/o **B. Control** Prenatal care n: 81/73 (randomized); (allocated/analyzed) Age: 30.1 (5.3) y/o	To evaluate the benefits of the M&B Program on the psychological health outcomes of postnatal women. Guideline: not specified for the intervention, Assessment instrument: EPDS for postnatal depressive symptoms Cutoff/eligibility criterion: EPDS ≥ 13 was used to identify women at risk for postnatal depression; not used as an eligibility criterion. Women receiving psychiatric care or requiring hospitalization were excluded. Some participants had EPDS ≥ 13 at baseline. Classification: prevention-oriented trial based on recruitment criteria; not selected for depressive symptoms, although not strictly asymptomatic at baseline.	Frequency: 1 once/8 weeks Group exercise (1once per weeks), and booklet for conducting of exercises at home.	Postpartum depression There is reduced in the EPDS scores between baseline and 8th weeks´. However, this effect was not observed between 8 and 12 weeks.	**A.** Intervention Baseline 8 weeks´ 12 weeks´	**A.** 8 (6.16) 5.47 (5.11) 4.73 (5.27) *n* = 62
			Intensity: not specified		**B.** Control Baseline 8 weeks´ 12 weeks´	**B.** 6.75 (5.44) 6.75 (5.51) 6.54 (5.61) *n* = 73
			Physical activity modality/classification: exercise with baby (cardiovascular and strength components)/structured exercise;			
			Professional assistance: physical therapist			
			Duration: 60 min			
Perales et al., 2015a,2015b, Spain/7	**A. Intervention** General care and exercise program n: 129 (randomized); n: 55/52 (allocated/analyzed) Age: 31.98 (3.74) y/o **B. Control** General care n: 64/54 (randomized); (allocated/analyzed) Age: 33.39 (3.98) y/o	To assess the effectiveness of a regular physical exercise program on depression and gestational weight gainin overweight and obese pregnant women. Guideline: ACOG Assessment instrument: CES-D Cutoff/eligibility criterion: CES-D ≥ 16 was considered indicative of depression; not used as an eligibility criterion. Depression was assessed in the first and third trimesters, and some participants had CES-D ≥ 16 at baseline. Classification: prevention-oriented trial based on recruitment criteria; not selected for depressive symptoms, although not strictly asymptomatic at baseline.	Frequency: 3 times per week/(28–30 weeks)	Antenatal depression The CES-D scores lowest in the third trimester for the exercise group, and for subgroups, included of the overweight and adequate weight gain.	**A.** Intervention 3rd trimester **B.** Control 3rd trimester	**A.** 9.33 (7.17) *n* = 45 **B.** 15.26 (9.76) *n* = 53
			Intensity: “fairly light” to “somewhat hard”			
			Physical activity modality/classification: warm-up - (aerobic activities), static stretching and in relaxing exercise (static stretching (pelvic floor muscle training, and partner massages)/structured exercise;			
			Professional assistance: fitness specialist and obstetrician’s assistance			
			Duration: 55–60 min			
Songøygard et al. 2012; Norway/8	**A. Intervention** n: 855 (randomized); n: 429/379 (allocated/analyzed) Age: >18 y/o 30.59 (4.3) y/o **B. Control** Regular antenatal care n: 426/340 (randomized); (allocated/analyzed) Age: >18 y/o 32.57 (4.2) y/o	To assessing if the regular exercise during pregnancy reduces the risk of postnatal depression Guideline: ACOG Assessment instrument: EPDS for postnatal depressive symptoms Cutoff/eligibility criterion: EPDS ≥ 10 was used to indicate probable minor depression and EPDS ≥ 13 to indicate probable major depression at 3 months postpartum; not used as eligibility criteria. Classification: prevention-oriented trial.	Frequency: 1 once/12-week; Home exercise (at leat twice week) of 45 min/structured exercise;	Postpartum depression There was no difference in total EPDS scores between the intervention and the control group.	**A.** Intervention 3 months (postpartum) **B.** Control 3 months (postpartum)	**A.** 2.52 (2.9) *n* = 379 **B.** 2.52 (3.3) *n* = 340
			Intensity: moderate to high			
			Physical activity modality/classification: aerobic activity, specific exercises (stabilization of the lower back and pelvis, pelvic floor muscles) and balance exercises. Home exercise: endurance training, strength/balance exercises/structured exercise;			
			Professional assistance: physiotherapists			
			Duration: 60 min			
**Symptomatic/treatment-oriented trials: women recruited with depression/anxiety diagnosis**,** elevated symptoms**,** at-risk status**,** or active treatment**						
Heh et al. 2008; Taiwan/3 **†**	A. **Intervention** n: 80 (randomized); n: 40/33 (allocated/analyzed) Age: 20 to 35 y/o B. **Control** Support care n: 40/30 (randomized); (allocated/analyzed) Age: 20 to 35 y/o	To examine the effectiveness of an exercise support program on reducing psychological morbidity after childbirth Guideline: not specified Assessment instrument: EPDS Cutoff/eligibility criterion: EPDS > 10/≥10 at 4 weeks postpartum; women were considered at risk of postnatal depression. Classification: symptomatic/at-risk trial based on screening cutoff; no formal clinical diagnosis reported.	Frequency: 3 times a week/3 months 1 session at hospital per week 2 session at home Intensity: low-intensity Physical activity modality/classification: physical activity mixed (45 min of stretching, 5 min of warm-up and 10 min of cool-down)/structured exercise; Professional assistance: not specified Duration: 60 min	Postpartum depression Women who received the exercise support program were less likely to have high depression scores after childbirth when compared to the control group.	**A**. Intervention 4th week PPD 20th week PPD . Control 4th week PPD 20th week PPD	**A**. 16.5 (2.6) 10.2 (3.6) *n* = 33 **B.** 16.3 (3.2) 12.7 (3.9) *n* = 30
Daley et al. 2015; UK/7 **†**	**A. Intervention** n: 94 (randomized); n: 47/41 (allocated/analyzed) Age: ≥18 y/o 31.7 (5.3) y/o **B. Control** Usual care n: 47/38 (randomized); (allocated/analyzed) Age: ≥18 y/o 29.3 (5.7) y/o	The participants receiving the exercise intervention would report lower depressive symptom scores at follow-up than those receiving usual care. Guideline: not specified for the intervention; Assessment instrument: EPDS for depressive symptoms; CIS-R for ICD-10 diagnosis of major depressive episode Cutoff/eligibility criterion: EPDS ≥ 10 used for initial screening, EPDS ≥ 13 used for second screening, followed by CIS-R confirmation of ICD-10 major depressive episode. Women with mixed anxiety and depression were also eligible. Classification: symptomatic/treatment-oriented trial; not prevention-oriented.	Frequency: 1–12 weeks: 3 times a week 13–24 weeks: 3–5 times per week	Postpartum depression Adjusting for baseline, there is − 2.04-(CI95%: −4.11 to 0.03) point mean difference in EPDS score at 6 months in the intervention group.	**A.** Intervention (postpartum) Baseline 6 months 12 months **B.** Control Baseline 6 months 12 months	**A.** 17.3 (3); *n* = 47 12.51 (5.46); *n* = 43 12.02 (5.29); *n* = 41 **B.** 17.5 (3.7); *n* = 47 14.67 (4.86); *n* = 42 12.55 (5.17); *n* = 38
			Intensity: moderate			
			Physical activity modality/classification: exercise modalities (stepping, cycling and walking)/structured exercise;			
			Professional assistance: personalized exercise consultations (1 and 2 months) and telephone calls (3 and 4 months)			
			Duration: 30 min			
Buttner et al. 2015; USA/6 **†**	**A. Intervention** n: 57 (randomized); n: 28/23 (allocated/analyzed) Age: 18–45 y/o 29.81 (5.17) y/o **B. Control** No intervention n: 29/27 (randomized); (allocated/analyzed) Age: 18–45 y/o 32.45 (4.78) y/o	To examine the efficacyof yoga for PPD and anxiety. Guideline: not specified Assessment instrument: PHQ-9 for screening; SCID-I for DSM-IV psychiatric assessment; HDRS **(1)** for depressive symptom severity; IDAS **(2)** for depressive and anxiety symptoms Cutoff/eligibility criterion: PHQ-9 ≥ 10 used for initial screening and HDRS ≥ 12 required for eligibility. SCID-I was used to assess DSM-IV psychiatric disorders. Women with anorexia, bipolar disorder, personality disorders, psychosis, or poorly controlled medical conditions were excluded. Classification: symptomatic/treatment-oriented trial; not prevention-oriented.	Frequency: 16 classes/8 weeks Practice at home (at least once a week from DVD video) of 30-min yoga/recreation;	Postpartum depression and anxiety Depressive symptoms decreased systematically over time. There is steeper linear decline in scores on the IDAS social anxiety	**A**. Intervention Pre-treatment	**1 A.** 17.33 (5.1) **2 A.** 10.96 (3.33) *n* = 23
			Intensity: not specified		2 weeks	**1 A.** 10.37 (5.36) **2 A.** 9.00 (3.14) *n* = 23
			Physical activity modality/classification: yoga (sun salutations, balancing, twisting, and relaxation poses)/recreation;		4 weeks	**1 A.** 8.77 (6.77) **2 A.** 8.87 (3.48) *n* = 23
			Professional assistance: yoga instructors		6 weeks	**1 A.** 6.35 (5.22) **2 A.** 7.71 (1.99) *n* = 23
			Duration: 1 h		8 weeks (post-treatment)	**1 A.** 5.87 (6.03) **2 A.** 7.00 (2.03) *n* = 23

**Abbreviations**: ACSM: American College of Sports Medicine; ACOG: American College of Obstetricians and Gynecologists; PPD: postpartum depression; CES-D: Center for Epidemiological Studies of Depression; EPDS: Edinburgh Postnatal Depression Scale; n: sample size; IDAS: Inventory of Depression and Anxiety Symptoms; HDRS: Hamilton Depression Rating Scale; POMS: profile of mood states; S-AI: State Anxiety Inventory; SD: standard deviation; y/o: years-old

aData in median (range)

bData in median (interquartile range)

† Symptomatic/treatment-oriented trial; analyzed separately from prevention-oriented trials

Cutoff points are presented when reported or used by the original trial for eligibility or outcome classification. Trials were classified as prevention-oriented when participants were not recruited on the basis of a baseline diagnosis of depression or anxiety, elevated depressive or anxiety symptoms, at-risk status, or active treatment. Trials enrolling symptomatic, at-risk, or actively treated participants at baseline were classified as symptomatic/treatment-oriented trials and analyzed separately from prevention-oriented trials. When at least three studies were available for the same outcome, these trials were pooled in a separate exploratory meta-analysis; otherwise, they were summarized narratively.

The included trials reported heterogeneous physical activity interventions, with variation in modality, supervision, frequency, session duration, program duration, and intensity reporting. Several studies reported adherence to guidelines from the American College of Obstetricians and Gynecologists (ACOG) (Robledo-Colonia et al. 2012; Perales et al. 2015a, 2015b; Perales et al. 2016; Aguilar-Cordero et al. 2019; Taniguchi and Sato 2016; Saccone et al. 2026), the American College of Sports Medicine (ACSM) (Coll et al. 2019; Navas et al. 2021), or the Guidelines on Sports for Pregnancy (Taniguchi and Sato 2016), with some studies referencing more than one guideline. Other trials did not specify whether the intervention was based on a particular guideline (Heh et al. 2008; Mohammadi et al. 2015; Rong et al. 2021; Norman et al. 2010; Daley et al. 2015; Buttner et al. 2015; Avery et al. 2026). Physical activity intensity was quantified or described in some studies, including through the Borg scale or predefined intensity categories (Robledo-Colonia et al. 2012; Perales et al. 2015a, 2015b; Perales et al. 2016; Aguilar-Cordero et al. 2019; Coll et al. 2019; Navas et al. 2021; Saccone et al. 2026). However, several studies did not clearly report the method used to quantify or monitor physical activity intensity (Heh et al. 2008; Mohammadi et al. 2015; Rong et al. 2021; Norman et al. 2010; Songøygard et al. 2012; Taniguchi and Sato 2016; Daley et al. 2015; Buttner et al. 2015; Avery et al. 2026).

Depressive symptoms were most frequently assessed using the Edinburgh Postnatal Depression Scale (EPDS) (Heh et al. 2008; Mohammadi et al. 2015; Aguilar-Cordero et al. 2019; Coll et al. 2019; Navas et al. 2021; Rong et al. 2021; Norman et al. 2010; Songøygard et al. 2012; Daley et al. 2015; Saccone et al. 2026; Avery et al. 2026). Additional instruments included the Center for Epidemiologic Studies Depression Scale (CES-D) (Robledo-Colonia et al. 2012; Perales et al. 2015a, 2015b; Perales et al. 2016), the Hamilton Depression Rating Scale (HDRS) (Buttner et al. 2015), and the Profile of Mood States (POMS) (Taniguchi and Sato 2016). Anxiety symptoms were assessed using the State Anxiety Inventory or State-Trait Anxiety Inventory (Rong et al. 2021; Avery et al. 2026), the Inventory of Depression and Anxiety Symptoms (IDAS) (Buttner et al. 2015), and the Profile of Mood States (POMS) (Taniguchi and Sato 2016).

### Quality assessment

The total PEDro score ranged from three to eight, with a median score of 6 (interquartile range: 5 to 7) (Table 1). No study reported concealed allocation. Additionally, due to the nature of the interventions, none of the trials blinded participants and/or therapists. All studies met the criteria for reporting statistical information about the trials.

### Summary of data from prevention-oriented trials

Among prevention-oriented trials, physical activity was associated with lower depressive symptom scores across the perinatal period, with favorable effects observed in both antenatal and postpartum analyses among women not recruited on the basis of depression/anxiety diagnosis, elevated symptoms, at-risk status, or active treatment at baseline. For antenatal anxiety symptoms, evidence was available from three trials and did not show a significant effect of physical activity compared with no intervention. These findings should be interpreted cautiously because of methodological limitations, variability across interventions and populations, and the low or very low certainty of evidence. The quantitative syntheses were interpreted as exploratory summaries complementing the systematic review, rather than as definitive pooled estimates of a single underlying intervention effect.

#### Antenatal depression symptoms

**Fig. 2:**
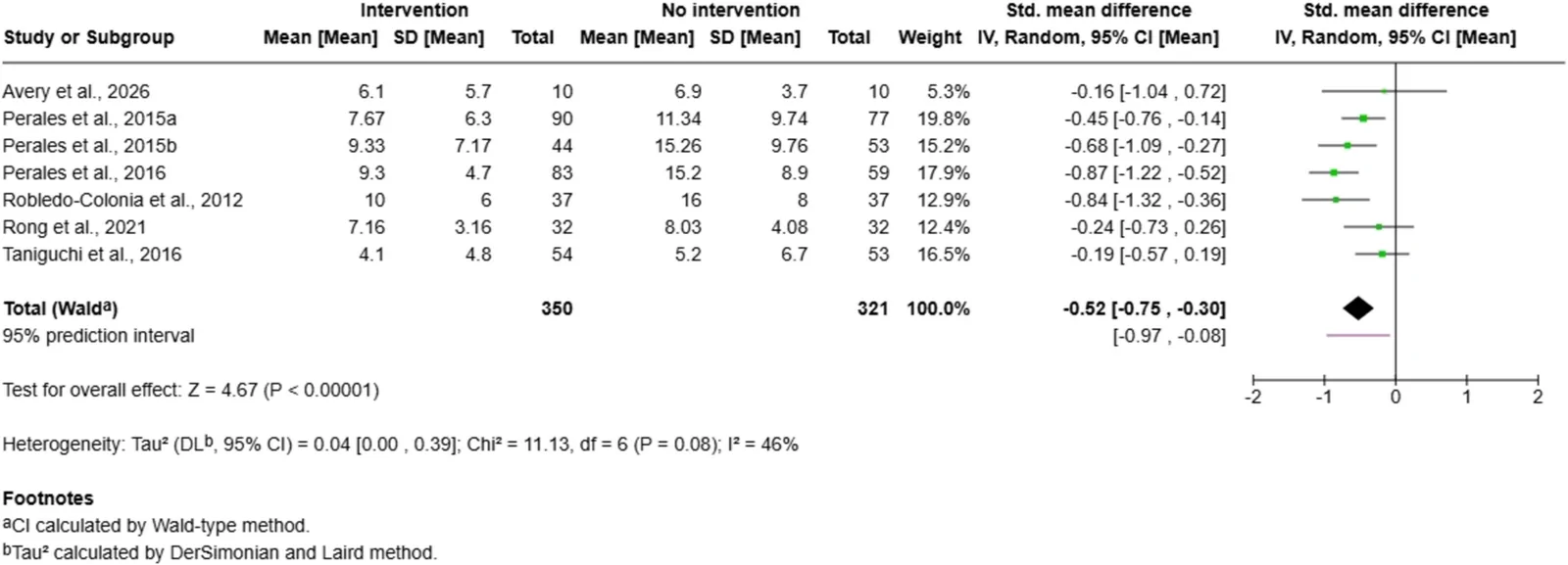
Impact of physical activity on antenatal depressive symptoms compared with no intervention in prevention-oriented trials

Seven RCTs (*n* = 671) assessed the effect of physical activity on antenatal depressive symptoms in prevention-oriented trials, in which women were not recruited on the basis of depression/anxiety diagnosis, elevated symptoms, at-risk status, or active treatment at baseline. Based on low-certainty evidence, physical activity was associated with lower antenatal depressive symptom scores compared with no intervention (SMD: −0.52, 95% CI: −0.75 to −0.30; I² = 46%; Fig. 2). The 95% prediction interval ranged from − 0.97 to −0.08, suggesting that the direction of effect may remain favorable across future comparable settings, although the magnitude of the effect may vary and the certainty of evidence was low.

#### Postpartum depressive symptoms

**Fig. 3:**
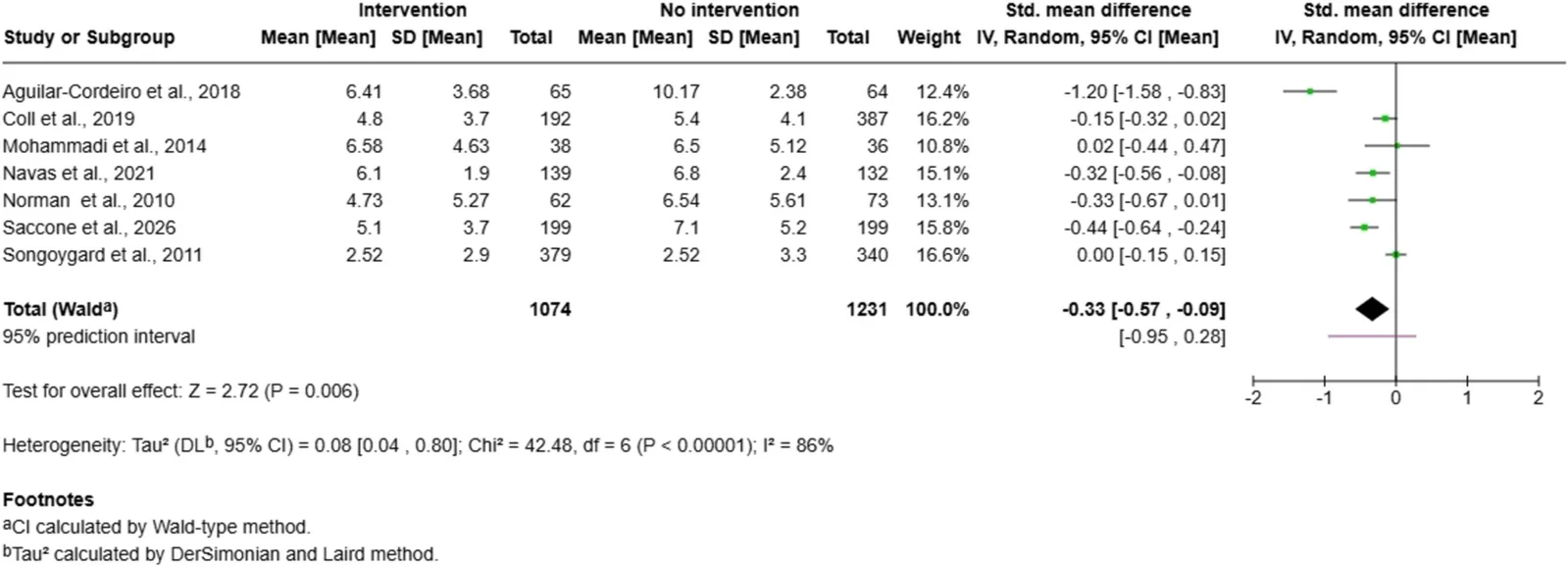
Impact of physical activity on postpartum depressive symptoms compared with no intervention in prevention-oriented trials

Seven RCTs (*n* = 2,305) assessed the effect of physical activity on postpartum depressive symptoms in prevention-oriented trials, in which women were not recruited on the basis of depression/anxiety diagnosis, elevated symptoms, at-risk status, or active treatment at baseline. Based on very low-certainty evidence, physical activity was associated with lower postpartum depressive symptom scores compared with no intervention (SMD: −0.33, 95% CI: −0.57 to −0.09; I² = 86%; Fig. 3). The 95% prediction interval ranged from − 0.95 to 0.28, suggesting that although the pooled estimate favored physical activity, the magnitude and direction of the effect may vary across future comparable settings.

#### Antenatal anxiety symptoms

**Fig. 4:**
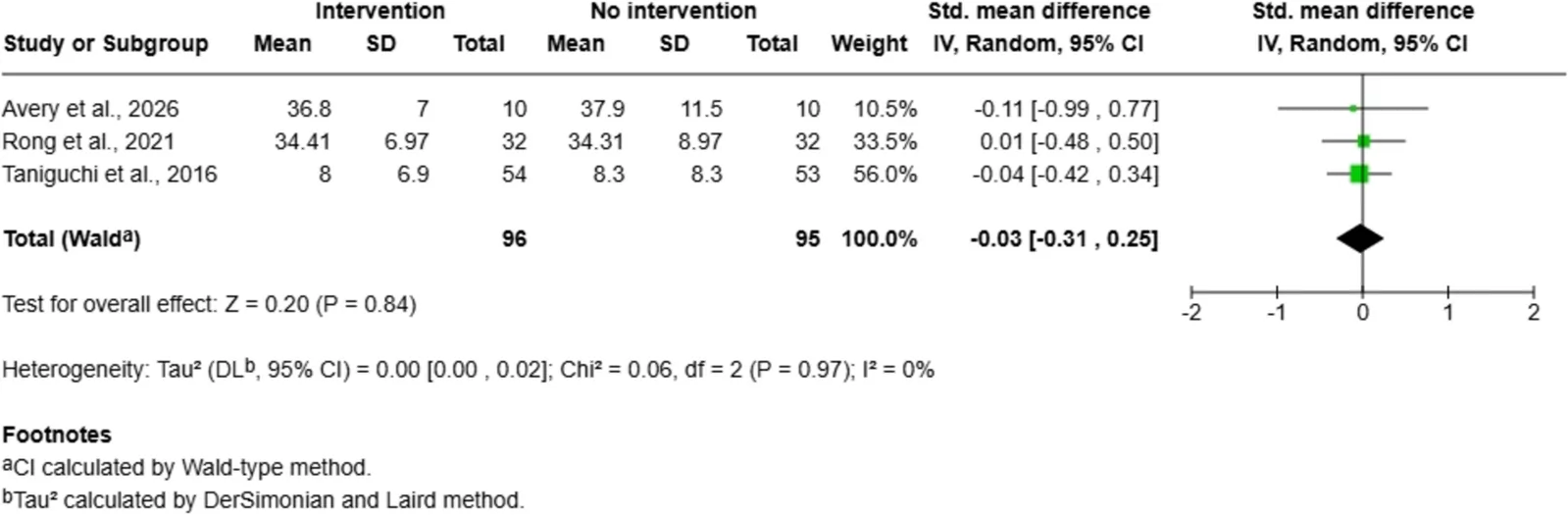
Impact of physical activity on antenatal anxiety symptoms compared with no intervention in prevention-oriented trials

Three RCTs (*n* = 191) assessed the effect of physical activity on antenatal anxiety symptoms in prevention-oriented trials, in which women were not recruited on the basis of depression/anxiety diagnosis, elevated symptoms, at-risk status, or active treatment at baseline. Based on very low-certainty evidence, physical activity was not associated with lower antenatal anxiety symptom scores compared with no intervention (SMD: −0.03, 95% CI: −0.31 to 0.25; I² = 0%; Fig. 4).

### Summary of data from symptomatic/treatment-oriented trials

Three trials enrolled women with elevated depressive symptoms, at-risk status, diagnosis, or active treatment at baseline and were therefore analyzed separately from prevention-oriented trials. In this subgroup, physical activity was associated with lower postpartum depressive symptom scores compared with no intervention, but the certainty of evidence was very low and the estimate should be interpreted cautiously because of methodological limitations, imprecision, and clinical heterogeneity. These findings should not be interpreted as definitive evidence of treatment efficacy, but rather as exploratory evidence suggesting that further trials specifically designed for symptomatic or treatment-oriented populations are needed.

#### Postpartum depressive symptoms

**Fig. 5:**
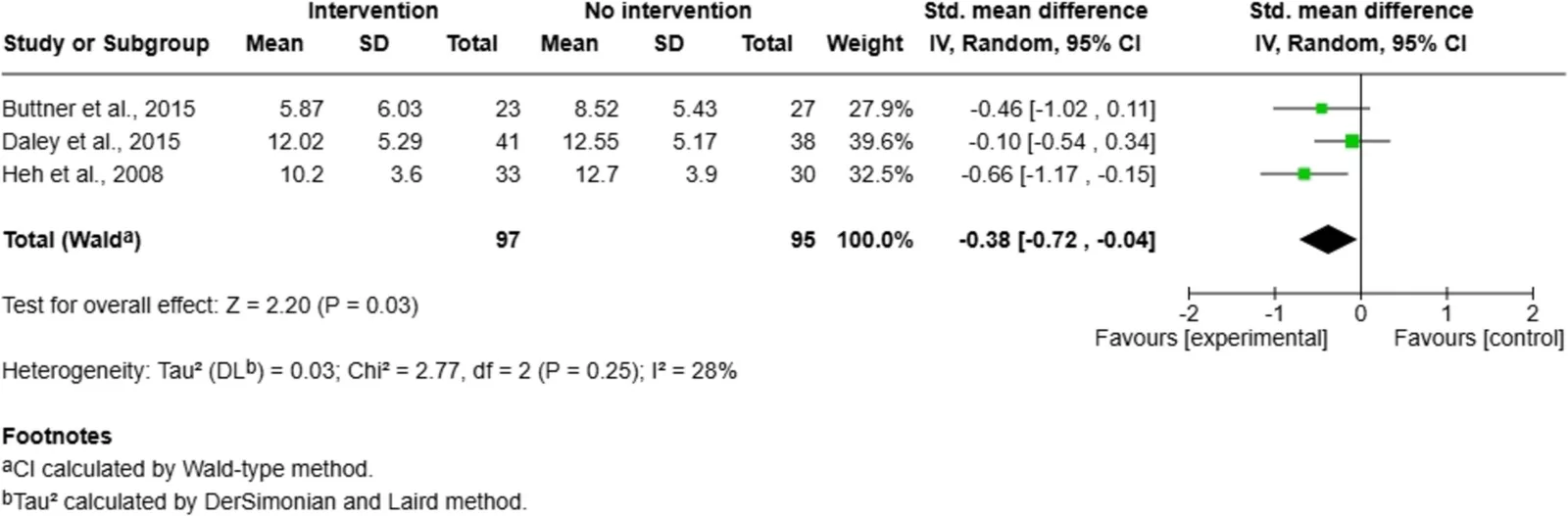
Impact of physical activity on postpartum depressive symptoms compared with no intervention in symptomatic/treatment-oriented trials

Three RCTs (*n* = 192) assessed the effect of physical activity on postpartum depressive symptoms in symptomatic/treatment-oriented trials. Based on very low-certainty evidence, physical activity was associated with lower postpartum depressive symptom scores compared with no intervention (SMD: −0.38, 95% CI: −0.72 to −0.04; I² = 28%; Fig. 5). These findings should be interpreted cautiously because of the small number of trials, methodological limitations, imprecision, and clinical heterogeneity across symptomatic or treatment-oriented populations.

#### Secondary outcomes

Secondary outcomes and exploratory subgroup analyses according to physical activity modality/classification, frequency/session duration, and intensity are presented in Supplementary Figures S1–S5. These analyses were restricted to prevention-oriented trials and should be interpreted cautiously because of the small number of studies in several subgroups, clinical and methodological heterogeneity, and low or very low certainty of evidence.

#### Antenatal depression symptoms (type exercise)

Five RCTs (*n* = 588) assessed the effect of structured physical activity on antenatal depressive symptoms in prevention-oriented trials. Based on low-certainty evidence, structured physical activity was associated with lower antenatal depressive symptom scores compared with no intervention (SMD: −0.59, 95% CI: −0.84 to −0.34; I² = 54%; Supplementary Figure S1). The 95% prediction interval ranged from − 1.07 to −0.11, suggesting that the direction of effect may remain favorable across future comparable settings, although the certainty of evidence was low.

#### Postpartum depression symptoms (type exercise)

Six RCTs (*n* = 2,034) assessed the effect of structured physical activity on postpartum depressive symptoms in prevention-oriented trials. Based on very low-certainty evidence, structured physical activity was associated with lower postpartum depressive symptom scores compared with no intervention (SMD: −0.34, 95% CI: −0.62 to −0.06; I² = 88%; Supplementary Figure S2). The 95% prediction interval ranged from − 1.03 to 0.35, suggesting that although the pooled estimate favored structured physical activity, the magnitude and direction of the effect may vary across future comparable settings.

#### Antenatal depression symptoms (frequency/time)

Five RCTs (*n* = 545) evaluated physical activity programs performed three times per week for approximately 60 min on antenatal depressive symptoms in prevention-oriented trials. Based on low-certainty evidence, these programs were associated with lower antenatal depressive symptom scores compared with no intervention (SMD: −0.62, 95% CI: −0.84 to −0.40; I² = 37%; Supplementary Figure S3). The 95% prediction interval ranged from − 0.99 to −0.25, suggesting that the direction of effect may remain favorable across future comparable settings, although the certainty of evidence was low.

#### Postpartum depression symptoms (frequency/time)

Three RCTs (*n* = 1,106) evaluated physical activity programs performed three times per week for approximately 60 min on postpartum depressive symptoms in prevention-oriented trials. Based on very low-certainty evidence, these programs were associated with lower postpartum depressive symptom scores compared with no intervention (SMD: −0.57, 95% CI: −1.05 to −0.09; I² = 92%; Supplementary Figure S4). These findings should be interpreted cautiously because of the small number of trials, substantial heterogeneity, and very low certainty of evidence.

#### Postpartum depression symptoms (moderate intensity)

Three RCTs (*n* = 979) evaluated the effect of moderate-intensity physical activity on postpartum depressive symptoms assessed using the EPDS in prevention-oriented trials. Based on very low-certainty evidence, moderate-intensity physical activity was associated with lower postpartum depressive symptom scores compared with no intervention (MD: −1.61, 95% CI: −3.16 to −0.07; I² = 93%; Supplementary Figure S5). Because this analysis used the same instrument across studies, the effect is presented as a mean difference on the EPDS scale; however, the result should be interpreted cautiously because of substantial heterogeneity and very low certainty of evidence.

#### Sensitivity analyses

Sensitivity analyses were conducted by excluding trials with lower methodological quality, defined as PEDro scores < 6. For postpartum depressive symptoms in prevention-oriented trials, exclusion of the trial with PEDro < 6 yielded an effect estimate consistent with the main analysis (SMD: −0.37, 95% CI: −0.67 to −0.07; I² = 88%; 95% prediction interval: −1.11 to 0.37). A similar pattern was observed for structured physical activity on postpartum depressive symptoms, with the pooled estimate remaining in favor of structured physical activity after exclusion of the lower-quality trial (SMD: −0.39, 95% CI: −0.76 to −0.01; I² = 90%; 95% prediction interval: −1.25 to 0.48). Overall, the direction of effect remained consistent with the primary analyses, although heterogeneity remained substantial and prediction intervals included the null.

## Discussion

This systematic review and meta-analysis synthesized randomized controlled trials evaluating isolated physical activity interventions compared with usual care or no intervention for depressive and anxiety symptoms during the perinatal period. In prevention-oriented trials, physical activity was associated with lower antenatal and postpartum depressive symptom scores, although the certainty of evidence was low for antenatal depression and very low for postpartum depression. No clear association was observed for antenatal anxiety symptoms. In a separate exploratory synthesis of symptomatic/treatment-oriented trials, physical activity was also associated with lower postpartum depressive symptom scores, but this finding was based on very low-certainty evidence and should be interpreted cautiously.

These findings underscore the potential role of physical activity in perinatal mental health care, while also highlighting the need for cautious interpretation. Another meta-analysis (Carter et al. 2019) examined the effectiveness of exercise-based interventions in preventing or treating postpartum depression among both first-time and experienced mothers up to 52 weeks postpartum. Most interventions focused on aerobic exercises and coaching compared with standard care. Although the results showed a modest reduction in depressive symptoms with exercise interventions, the certainty of evidence across the analyzed studies was consistently low (Schünemann et al. 2013a, 2013b).

Accordingly, the pooled estimates should be interpreted as exploratory rather than confirmatory. Although random-effects models were used to account for between-study variability, substantial heterogeneity persisted in several analyses, particularly for postpartum depressive symptoms. The prediction intervals included the null in some analyses, indicating that the effect of physical activity may vary across future comparable settings and may be small or absent in some contexts. Sensitivity analyses excluding trials with PEDro scores < 6 yielded estimates consistent with the main analyses, but did not materially reduce heterogeneity. These findings reinforce the need for cautious interpretation, particularly given the low or very low certainty of evidence. The separate analysis of symptomatic/treatment-oriented trials is an important addition to the interpretation of the evidence. These trials enrolled women with elevated depressive symptoms, at-risk status, diagnosis, or active treatment at baseline and were therefore not combined with prevention-oriented trials. In this subgroup, physical activity was associated with lower postpartum depressive symptom scores compared with no intervention. However, because this synthesis included only three trials and the certainty of evidence was very low, the finding should be interpreted as hypothesis-generating rather than as definitive evidence of treatment efficacy. Separating these trials from prevention-oriented analyses was methodologically important because baseline symptom profile may influence both the magnitude of response and the clinical interpretation of physical activity interventions.

From a clinical perspective, the standardized mean differences (SMDs) should be interpreted cautiously. Because the included trials used different instruments to assess depressive symptoms and differed in baseline symptom severity, timing of assessment, and population characteristics, we did not back-transform pooled SMDs into a single raw-score metric such as EPDS points. Although such conversions can be useful when a representative standard deviation is available, in this review they could be misleading because no single instrument or baseline variability adequately represented all pooled studies. Therefore, the clinical interpretation was based primarily on the magnitude and direction of the standardized effects, prediction intervals, and the certainty of evidence. Small-to-moderate standardized effects may still be clinically relevant at the population level, particularly for women with mild or borderline symptoms; however, the pooled SMDs cannot be used to determine whether participants crossed diagnostic or screening thresholds such as EPDS ≥ 10 or ≥ 13. Very large estimates, particularly in sparse antenatal analyses, should be interpreted with caution, as they may reflect limited data and between-study heterogeneity rather than a stable intervention effect.

When outcomes were reported using the same scale, as in the supplementary analysis of moderate-intensity physical activity on postpartum depressive symptoms using the EPDS, mean differences were interpreted directly in raw-score units. However, this interpretation was restricted to that specific analysis and was not generalized to pooled SMD analyses based on different instruments.

Structured physical activity programs may plausibly be associated with more consistent effects than less structured or recreational approaches because they tend to provide clearer exercise prescriptions, regular supervision, progression criteria, adherence support, and greater consistency in frequency, intensity, and duration. Similarly, programs delivered three times per week for approximately 60 min may provide a sufficient exercise dose and behavioral routine to influence depressive symptoms through physiological and psychosocial pathways. However, these subgroup findings should be considered exploratory and hypothesis-generating rather than definitive. The confidence intervals were wide in several analyses, prediction intervals often included the null, and the certainty of evidence was low or very low. Therefore, the apparent differences across intervention characteristics should not be interpreted as proof that one specific exercise format, frequency, duration, or intensity is superior.

Our results were inconclusive in evaluating the effects of physical activity on anxiety. In the revised analysis, three prevention-oriented trials involving pregnant women were included in the quantitative synthesis (Avery et al. 2026; Rong et al. 2021; Taniguchi and Sato 2016). The pooled estimate did not show a clear reduction in anxiety symptoms among women who participated in physical activity programs compared with those in the no-intervention group (SMD: −0.03, 95% CI: −0.31 to 0.25; I² = 0%). Given the small number of trials and the imprecision of the estimate, this finding should be interpreted as insufficient evidence rather than evidence that physical activity is ineffective for anxiety symptoms during pregnancy.

The positive effects of regular physical activity in reducing depression and depressive symptoms can be attributed to both physiological and psychological mechanisms (Xie et al. 2021). Physical activity has been shown to increase the concentrations of neurotransmitters such as 5-hydroxytryptamine, dopamine, and noradrenaline, which contribute to alleviating depressive symptoms (Jaromin et al. 2019; Lin and Kuo 2013). Additionally, serotonin, another key neurotransmitter, becomes more available through physical activity, facilitating mood regulation and modulating chronic stress responses via the hypothalamic–pituitary–adrenal (HPA) axis. This process helps reduce symptoms of depression and anxiety (Singh et al. 2023). The presence of depression and anxiety is associated with dysfunction of the HPA axis, resulting in its hyperactivation and subsequent cortisol overproduction, which underlies physiological stress responses (Strawbridge et al. 2017; Lamers et al. 2019).

From a psychological perspective, engaging in physical activity enhances self-esteem, boosts self-confidence, and improves overall satisfaction and quality of life (Rimer et al. 2012; Schuch et al. 2016). Additionally, regular physical activity has been shown to increase the concentration of brain-derived neurotrophic factor (BDNF) (Arosio et al. 2021; Erickson et al. 2011), which is often reduced in individuals with depression. The improvement in depressive symptoms is partly attributed to the effects of BDNF on neurogenesis and brain neuroplasticity (Bjørnebekk et al. 2005; Phillips 2017; Dinoff et al. 2017). Furthermore, the pathogenesis of depression and anxiety is associated with neuroinflammation (Kandola et al. 2019; Eyre and Baune 2012). Physical activity contributes to the regulation of brain chemistry, which, in turn, helps mitigate inflammatory processes (Eyre and Baune 2012).

Physical activity has the potential to reduce inflammation and stimulate the release of vascular endothelial growth factor (VEGF), growth hormone (GH), and insulin-like growth factor 1 (IGF-1), thereby enhancing synaptic plasticity (Singh et al. 2023). These mediators, particularly GH and IGF-1, play a role in regulating sleep, cognitive function, and mood. Additionally, physical activity promotes the pro-neurogenic effects of progenitor cells in the hippocampus (Eyre and Baune 2012), which may contribute to improvements in depression and anxiety (Brooker et al. 2017; Duman and Aghajanian 2012; Cotman, Berchtold and Christie 2007).

Depression is closely linked to the production of inflammatory cytokines, which can be modulated by physical activity (American College of Obstetricians and Gynecologists 2018; Singh et al. 2023). Under conditions of chronic stress, immune cells are activated to secrete pro-inflammatory cytokines, potentially compromising the integrity of the blood–brain barrier. This disruption can lead to dysregulated signaling and symptoms commonly associated with depression, such as irritability, anhedonia, and depressed mood (Hassamal 2023). Furthermore, the dysregulation of pro-inflammatory markers, including IL-6, IL-1, TNF-α, and CRP, may contribute to reduced serotonin levels in the synaptic cleft. According to the monoaminergic theory, this reduction in serotonin availability is a key factor in the onset and progression of depression (Ruiz et al. 2022; Cui et al. 2024).

Although the evidence for anxiety outcomes was limited, a biological and psychosocial rationale remains plausible. Anxiety shares several characteristics with other mood disorders, including fear, chronic stress, and mood alterations (Leistner and Menke 2020). In the perinatal period, hormonal fluctuations, including changes in progesterone and estrogen levels and their influence on hypothalamic–pituitary–adrenal (HPA) axis responsiveness, may contribute to anxiety symptoms through altered stress responses and cortisol regulation (Navas-Arrebola et al. 2023; Anderson and Shivakumar 2013; Hinds and Sanchez 2022). Physical activity may plausibly influence anxiety symptoms by regulating cortisol levels, improving stress responses, enhancing mood and well-being, and reducing insomnia, fatigue, and exhaustion through neurochemical pathways involving endorphins, serotonin, dopamine, and noradrenaline (Jarbou and Newell 2022; Basso and Suzuki 2017; Anderson and Shivakumar 2013). However, because this rationale was not supported by robust trial evidence in the present review, no firm conclusions can be drawn regarding the effects of physical activity on perinatal anxiety.

Our results, which could inform the characteristics of a physical activity program with positive effects on depressive symptoms in the postpartum period, were constrained by the variability of interventions and the limited number of trials. Nonetheless, current recommendations for regular physical activity suggest engaging in moderate-intensity exercises for 30–60 min per session, with a frequency of 3–4 times per week during the postpartum period (American College of Obstetricians and Gynecologists 2020).

The strengths of this systematic review include the exclusive selection of randomized controlled trials, providing a robust assessment of the impact of interventions in a randomized and controlled manner. Furthermore, this review identifies components of physical activity programs that may be associated with greater reductions in depressive symptoms, although these subgroup findings should be interpreted as exploratory. These findings are consistent with current recommendations that encourage physical activity during the perinatal period, with careful consideration of individual needs, clinical status, and medical guidance.

However, this review also has several limitations that should frame the interpretation of the findings. First, important methodological limitations were common across the included trials. No study reported concealed allocation, and blinding of participants and therapists was not performed, which is expected in physical activity interventions but still increases the possibility of performance and detection bias. Second, the included trials varied substantially in diagnostic and symptom assessment tools, including instruments such as the EPDS, CES-D, and HDRS, which likely contributed to heterogeneity and limited direct clinical comparability across studies. Third, the interventions differed markedly in type, supervision, setting, duration, frequency, intensity, progression criteria, and mode of delivery. Fourth, sample sizes varied widely across studies, contributing to imprecision and limiting the stability of pooled estimates. In addition, baseline symptom profiles varied across trials. Some prevention-oriented trials did not recruit women on the basis of elevated symptoms or diagnosis but nevertheless included participants with elevated baseline scores, whereas other trials specifically enrolled symptomatic, at-risk, or actively treated women. Although these populations were analyzed separately, residual clinical heterogeneity related to baseline mental health status may have influenced the pooled estimates. These limitations reinforce that the findings should be interpreted as cautious, exploratory evidence rather than definitive estimates of intervention effectiveness.

### Implications for practice and research

High clinical and methodological heterogeneity and sparse data for anxiety limited the certainty of evidence; accordingly, pooled effects should be interpreted as indicative signals rather than definitive magnitudes. In particular, the lack of allocation concealment, absence of blinding, variability in outcome instruments, and diversity of intervention protocols reduce confidence in the precision and generalizability of the pooled estimates.

From a practical perspective, this study provides a synthesis of evidence supporting the integration of physical activity into clinical practice to reduce depressive symptoms during the perinatal period. Nonetheless, the certainty of evidence was low or very low, emphasizing the need for caution when interpreting the results.

Future research should focus on examining both the preventive role of physical activity during the perinatal period and its potential adjunctive role among women with elevated depressive symptoms, at-risk status, diagnosis, or active treatment at baseline. These studies should aim to clarify the effects of different intervention characteristics, including modality, frequency, intensity, and duration, on depression and anxiety outcomes. Additionally, future investigations should offer more comprehensive descriptions of data collection processes and methodologies, with a particular emphasis on participants’ physical activity history and practices before pregnancy. A detailed account of antidepressant and/or anxiolytic medication use prior to pregnancy is also recommended to improve the interpretability and practical application of research findings.

## Conclusion

The findings of this systematic review and meta-analysis suggest that isolated physical activity interventions during the antenatal and postpartum periods may be associated with reductions in depressive symptoms, particularly in prevention-oriented trials. A separate exploratory analysis of symptomatic/treatment-oriented trials also suggested lower postpartum depressive symptom scores among women receiving physical activity interventions compared with no intervention. However, the certainty of evidence was low to very low, and substantial clinical and methodological heterogeneity limits confidence in the magnitude and generalizability of the pooled estimates. Evidence regarding anxiety outcomes remains insufficient and inconclusive. Structured programs and interventions performed three to four times per week for approximately 30 to 60 min may be associated with greater reductions in depressive symptoms, but these subgroup findings should be interpreted as exploratory and hypothesis-generating rather than definitive evidence of an optimal exercise prescription. Further well-designed randomized controlled trials with larger sample sizes, clearer allocation procedures, standardized outcome assessment, and detailed reporting of intervention characteristics are needed to clarify the role of physical activity in preventing or reducing perinatal depression and anxiety symptoms.

## Supplementary Information

Below is the link to the electronic supplementary material.

Supplementary Material 1 (DOCX 17.4 KB)

Supplementary Material 2 (DOCX 21.4 KB)

Supplementary Material 3 (DOCX 22.0 KB)

Supplementary Material 4 (DOCX 28.7 KB)

Supplementary Material 5 (DOCX 314 KB)

## Data Availability

The data used in this systematic review were extracted from publicly available documents through their respective journals and databases. All data analyzed and their supplementary files during this study are included in this published document.

## Abbreviations

ACOG: The American College of Obstetricians and Gynecologists
ACSM: American College of Sports Medicine
BDNF: Brain: derived neurotrophic factor
CES-D: Center for Epidemiological Studies Depression Scale
CRP-C: reactive protein
EPDS: Edinburgh Postnatal Depression Scale
GH: Growth hormone
GRADE: Grading of Recommendations Assessment, Development and Evaluation
HDRS: Hamilton Depression Rating Scale
HPA: Hypothalamic-pituitary-adrenal
ICD: International Classification of Diseases
IDAS: Inventory of Depression and Anxiety Symptoms
IGF1: insulin-like growth factor
IL-6: interleukin 6
IL-1: interleukin 1
MD: Mean differences
PEDro: Physiotherapy Evidence Database
POMS: Profile of Mood States
PRISMA: Preferred Reporting Items for Systematic Reviews and Meta-Analysis
RCT: randomized controlled trial
S-AI: State Anxiety Inventory
SMD: standardized mean differences
TNF-α: Tumor necrosis factor alpha
VEGF: Vascular Endothelial Growth Factor
WHO: World Health Organization
