# Supplementary material for: Isolated physical activity programs during pregnancy and postpartum: Meta-analysis of randomized trials with usual-care controls on depressive and anxiety symptoms
Source: Arch Womens Ment Health. 2026 Sep 14;29(5):116. doi: 10.1007/s00737-026-01752-9 (PMC13572343; doi:10.1007/s00737-026-01752-9)
Supplement: Supplementary file 1 [file 737_2026_1752_MOESM1_ESM.docx]

**Supplement 1 - Search strategies. Divinópolis, Minas Gerais, Brazil.**

| **Database** | **Search strategy** |
| --- | --- |
| **PubMed** | ( "Pregnancy"[MeSH Terms] OR "Pregnant People"[MeSH Terms] OR "Prenatal Care"[MeSH Terms] OR "Postpartum Period"[MeSH Terms] OR gestatio*[Title/Abstract] OR pregnan*[Title/Abstract] OR "pregnant woman"[Title/Abstract] OR "pregnant women"[Title/Abstract] OR antenatal[Title/Abstract] OR prenatal[Title/Abstract] OR puerperium[Title/Abstract] ) AND ( "Exercise"[MeSH Terms] OR "Exercise Movement Techniques"[MeSH Terms] OR "Exercise Therapy"[MeSH Terms] OR "Physical Therapy Modalities"[MeSH Terms] OR "Physical Fitness"[MeSH Terms] OR "Physical Endurance"[MeSH Terms] OR "Sports"[MeSH Terms] OR "Tai Ji"[MeSH Terms] OR "Yoga"[MeSH Terms] OR "Qigong"[MeSH Terms] OR "balance training"[Title/Abstract] OR "functional training"[Title/Abstract] OR "aerobic activities"[Title/Abstract] OR "aerobic activity"[Title/Abstract] OR "aerobic exercise"[Title/Abstract] OR "aerobic training"[Title/Abstract] OR "cardiovascular activities"[Title/Abstract] OR "cardiovascular activity"[Title/Abstract] OR "endurance activities"[Title/Abstract] OR "endurance activity"[Title/Abstract] OR "endurance training"[Title/Abstract] OR "physical conditioning"[Title/Abstract] OR "resistance training"[Title/Abstract] OR "strength training"[Title/Abstract] OR "tai chi"[Title/Abstract] OR "tai ji"[Title/Abstract] OR yoga[Title/Abstract] OR walk[Title/Abstract] OR walking[Title/Abstract] OR "chi kung"[Title/Abstract] OR qigong[Title/Abstract] OR exercise*[Title/Abstract] OR "physical activity"[Title/Abstract] OR "physical activities"[Title/Abstract] OR sport*[Title/Abstract] ) AND ( "Depression"[MeSH Terms] OR "Depressive Disorder"[MeSH Terms] OR "Puerperal Disorders"[MeSH Terms] OR "Depression, Postpartum"[MeSH Terms] OR "depressive syndrome"[Title/Abstract] OR depression[Title/Abstract] OR "major depression"[Title/Abstract] OR "depressive symptoms"[Title/Abstract] OR "depressive symptom"[Title/Abstract] OR "depressive disorder"[Title/Abstract] OR "depressed mood"[Title/Abstract] OR "depressive state"[Title/Abstract] OR "depressive episode"[Title/Abstract] OR "Anxiety"[MeSH Terms] OR "Mental Disorders"[MeSH Terms] OR "Anxiety Disorders"[MeSH Terms] OR anxiety[Title/Abstract] ) AND ( "Randomized Controlled Trial"[Publication Type] OR "Controlled Clinical Trial"[Publication Type] OR randomi*ed[Title/Abstract] OR randomly[Title/Abstract] OR placebo[Title/Abstract] OR sham[Title/Abstract] OR "Clinical Trials as Topic"[MeSH Terms] OR trial[Title/Abstract] ) |
| **PEDro** | Depression (AB TI) AND Continence and Women’s health (Subdiscipline) AND Clinical trial (method) AND Exercise (TI) Depression (AB TI) AND Continence and Women’s health (Subdiscipline) AND Clinical trial (method) AND "Physical activity" (TI) Depression (AB TI) AND Strength training (Therapy) Continence and Women’s health (Subdiscipline) AND Clinical trial (method) Depression (AB TI) AND Fitness training (Therapy) Continence and Women’s health (Subdiscipline) AND Clinical trial (method) Depression (AB TI) AND Continence and Women’s health (Subdiscipline) AND Clinical trial (method) AND "Yoga" (TI) Depression (AB TI) AND Continence and Women’s health (Subdiscipline) AND Clinical trial (method) AND "Walk*" (TI) Depression (AB TI) AND Continence and Women’s health (Subdiscipline) AND Clinical trial (method) AND "Walk*" (TI) Depression (AB TI) AND Continence and Women’s health (Subdiscipline) AND Clinical trial (method) AND "Walk*" (TI) Depression (AB TI) AND Continence and Women’s health (Subdiscipline) AND Clinical trial (method) AND "Endurance" (TI) Depression (AB TI) AND Continence and Women’s health (Subdiscipline) AND Clinical trial (method) AND "Resistance" (TI) Depression (AB TI) AND Continence and Women’s health (Subdiscipline) AND Clinical trial (method) AND "Water" (TI) Anxiety (AB TI) AND Continence and Women’s health (Subdiscipline) AND Clinical trial (method) AND "Physical activity" (TI) Anxiety (AB TI) AND Continence and Women’s health (Subdiscipline) AND Clinical trial (method) AND "Exercise" (TI) Anxiety (AB TI) AND Strength training (Therapy) Continence and Women’s health (Subdiscipline) AND Clinical trial (method) Anxiety (AB TI) AND Fitness training (Therapy) Continence and Women’s health (Subdiscipline) AND Clinical trial (method) Anxiety (AB TI) AND Continence and Women’s health (Subdiscipline) AND Clinical trial (method) AND "Yoga" (TI) Anxiety (AB TI) AND Continence and Women’s health (Subdiscipline) AND Clinical trial (method) AND "Walk*" (TI) Anxiety (AB TI) AND Continence and Women’s health (Subdiscipline) AND Clinical trial (method) AND "Tai Chi" (TI) Anxiety (AB TI) AND Continence and Women’s health (Subdiscipline) AND Clinical trial (method) AND "Aerobic" (TI) Anxiety (AB TI) AND Continence and Women’s health (Subdiscipline) AND Clinical trial (method) AND "Endurance" (TI) Anxiety (AB TI) AND Continence and Women’s health (Subdiscipline) AND Clinical trial (method) AND "Resistance" (TI) Anxiety (AB TI) AND Continence and Women’s health (Subdiscipline) AND Clinical trial (method) AND "Water" (TI) |
| **EMBASE** | ('pregnant woman'/exp OR 'pregnant woman' OR 'prenatal care'/exp OR 'prenatal care' OR 'puerperium'/exp OR 'puerperium' OR 'postpartum period'/exp OR 'postpartum period' OR 'pregnancy'/exp OR 'pregnancy' OR 'gestation'/exp OR 'gestation' OR 'antenatal depression'/exp OR 'antenatal depression' OR antenatal OR 'prenatal'/exp OR 'prenatal') AND ('depression'/exp OR 'depression' OR 'depressive disorder'/exp OR 'depressive disorder' OR 'puerperal disorder'/exp OR 'puerperal disorder' OR 'postnatal depression'/exp OR 'postnatal depression' OR 'depression postpartum'/exp OR 'depression postpartum' OR 'major depression'/exp OR 'major depression' OR 'depressive symptoms'/exp OR 'depressive symptoms' OR 'depressed mood'/exp OR 'depressed mood' OR 'depressive state'/exp OR 'depressive state' OR 'depressive episode'/exp OR 'depressive episode' OR 'anxiety'/exp OR 'anxiety' OR 'anxiety disorder'/exp OR 'anxiety disorder' OR 'mental disease'/exp OR 'mental disease' OR 'mental disorder'/exp OR 'mental disorder') AND ('exercise' OR 'exercise movement techniques' OR 'exercise therapy' OR 'physical therapy modalities' OR 'physical fitness' OR 'endurance training' OR 'physical endurance' OR 'endurance' OR 'sports' OR 'tai chi' OR 'tai ji' OR 'yoga' OR 'balance training' OR 'qigong' OR 'qigong exercise' OR 'functional training' OR 'aerobic activity' OR 'aerobic exercise' OR 'cardiovascular activity' OR 'endurance activity' OR 'physical condition') AND ('randomized controlled trial' OR 'controlled clinical trial' OR random OR 'placebo' OR randomly OR 'clinical trial') AND [01-02-2024]/sd NOT [17-06-2026]/sd |
| **PsycINFO** | ( 'pregnant woman'/exp OR 'pregnant woman' OR 'prenatal care'/exp OR 'prenatal care' OR 'puerperium'/exp OR 'puerperium' OR 'postpartum period'/exp OR 'postpartum period' OR 'pregnancy'/exp OR 'pregnancy' OR 'gestation'/exp OR 'gestation' OR 'antenatal depression'/exp OR 'antenatal depression' OR antenatal OR 'prenatal'/exp OR 'prenatal' ) AND ( 'depression'/exp OR 'depression' OR 'depressive disorder'/exp OR 'depressive disorder' OR 'puerperal disorder'/exp OR 'puerperal disorder' OR 'postnatal depression'/exp OR 'postnatal depression' OR 'depression postpartum'/exp OR 'depression postpartum' OR 'major depression'/exp OR 'major depression' OR 'depressive symptoms'/exp OR 'depressive symptoms' OR 'depressed mood'/exp OR 'depressed mood' OR 'depressive state'/exp OR 'depressive state' OR 'depressive episode'/exp OR 'depressive episode' OR 'anxiety'/exp OR 'anxiety' OR 'anxiety disorder'/exp OR 'anxiety disorder' OR 'mental disease'/exp OR 'mental disease' OR 'mental disorder'/exp OR 'mental disorder' ) AND ( 'exercise' OR 'exercise movement techniques' OR 'exercise therapy' OR 'physical therapy modalities' OR 'physical fitness' OR 'endurance training' OR 'physical endurance' OR 'endurance' OR 'sports' OR 'tai chi' OR 'tai ji' OR 'yoga' OR 'balance training' OR 'qigong' OR 'qigong exercise' OR 'functional training' OR 'aerobic activity' OR 'aerobic exercise' OR 'cardiovascular activity' OR 'endurance activity' OR 'physical condition' ) AND ( 'randomized controlled trial' OR 'controlled clinical trial' OR random OR 'placebo' OR randomly OR 'clinical trial' ) |
| **SPORTDiscus** | DE "PREGNANCY" OR DE "PREGNANT women"  TI Pregnancy OR AB Pregnancy TI pregnan* OR AB pregnan DE "PUERPERIUM" TI gestatio* OR AB gestatio* TI Prenatal OR AB Prenatal TI matern* OR AB matern* TI antenatal OR AB antenatal TI puerperium OR AB puerperium S1 OR S2 OR S3 OR S4 OR S5 OR S6 OR S7 OR S8 OR S9  (((((((((((((((DE "EXERCISE") OR (DE "ABDOMINAL exercises")) OR (DE "AEROBIC exercises")) OR (DE "AQUATIC exercises")) OR (DE "CIRCUIT training")) OR (DE "EXERCISE therapy")) OR (DE "HATHA yoga")) OR (DE "MUSCLE strength")) OR (DE "PILATES method")) OR (DE "QI gong")) OR (DE "TAI chi")) OR (DE "TREADMILL exercise")) OR (DE "YOGA")) OR (DE "PHYSICAL activity")) OR (DE "PHYSICAL training & conditioning")) OR (DE "SPORTS") TI "Functional training" OR AB "Functional training" TI "Aerobic activities" OR AB "Aerobic activities" TI "Aerobic activity" OR AB "Aerobic activity" TI "Aerobic training" OR AB "Aerobic training" TI "Cardiovascular activities" OR AB "Cardiovascular activities" TI "Cardiovascular activity" OR AB "Cardiovascular activity" TI "Endurance activities" OR AB "Endurance activities" TI "Endurance activity" OR AB "Endurance activity" TI "Endurance training" OR AB "Endurance training" TI "Physical conditioning" OR AB "Physical conditioning" TI "Resistance training" OR AB "Resistance training" TI "strength training" OR AB "strength training" TI "Tai chi" OR AB "Tai chi" TI "Tai ji" OR AB "Tai ji" TI "Yoga" OR AB "Yoga" TI "Walk" OR AB "Walk" TI "Walking" OR AB "Walking" TI "Chi kung" OR AB "Chi kung" TI "Qigong" OR AB "Qigong" TI exercise* OR AB exercise* TX exercise TX "physical activity" TI ("physical activit*" or sport*) OR AB ("physical activit*" or sport*) S11 OR S12 OR S13 OR S14 OR S15 OR S16 OR S17 OR S18 OR S19 OR S20 OR S21 OR S22 OR S23 OR S24 OR S25 OR S26 OR S27 OR S28 OR S29 OR S30 OR S31 OR S32 OR S33 OR S34 (DE "MENTAL depression") OR (DE "ANXIETY") TI Depression OR AB Depression TI "Depressive Disorder" OR AB "Depressive Disorder" TI "depressive syndrome" OR AB "depressive syndrome" TI (major N2 depression) OR AB (major N2 depression) TI (Depressive N3 symptoms*) OR AB (Depressive N3 symptoms*) TI "(depressed N2 mood)" OR AB "(depressed N2 mood)" TI (depressed N3 mood) OR AB (depressed N3 mood) TI "depressive state" OR AB "depressive state" TI "depressive episode" OR AB "depressive episode" TI "depressive syndrome" OR AB "depressive syndrome" TI Anxiety OR AB Anxiety TI ( mental disorders or anxiety disorders ) OR AB ( mental disorders or anxiety disorders ) TX depression TX anxiety S36 OR S37 OR S38 OR S39 OR S40 OR S41 OR S42 OR S43 OR S44 OR S45 OR S46 OR S47 OR S48 OR S49 OR S50 TI trial OR AB trial AB randomly "controlled N 2 trial" OR "clinical trial" random* "random sampling" clinic* W3 trial* "random allocation" randomized OR randomised TX "controlled trials"  TX "controlled trial" TX "Clinical trial" S52 OR S53 OR S54 OR S55 OR S56 OR S57 OR S58 OR S59 OR S60 OR S61 OR S62 S10 AND S35 AND S51 AND S63 |

Note: Searches were initially conducted in February 2022 and updated in February 2024. An additional update was conducted on 14 June 2026, covering records published or indexed from February 2024 to June 2026. The same conceptual search strategy and eligibility criteria were maintained. Database-specific syntax, descriptors, field tags, and parentheses were checked and adapted when necessary. No language restrictions were applied.
