# Supplementary material for: Isolated physical activity programs during pregnancy and postpartum: Meta-analysis of randomized trials with usual-care controls on depressive and anxiety symptoms
Source: Arch Womens Ment Health. 2026 Sep 14;29(5):116. doi: 10.1007/s00737-026-01752-9 (PMC13572343; doi:10.1007/s00737-026-01752-9)
Supplement: Supplementary file 2 [file 737_2026_1752_MOESM2_ESM.docx]

*Supplement 2* - Levels of quality evidence (GRADE)

| **Certainty assessment** | | | | | | | **№ of patients** | | **Effect** | | **Certainty** | **Importance** |
| --- | --- | --- | --- | --- | --- | --- | --- | --- | --- | --- | --- | --- |
| **№ of studies** | **Study design** | **Risk of bias** | **Inconsistency** | **Indirectness** | **Imprecision** | **Other considerations** | **physical activity** | **no intervention** | **Relative (95% CI)** | **Absolute (95% CI)** |  |  |
| Figure 2. Antenatal depressive symptoms in prevention-oriented trials | | | | | | | | | | | | |
| 7 | Randomized clinical trials | Serious^a^ | Not serious | Not serious | Not serious | Publication bias strongly suspected^c^ | 350 | 321 | - | (SMD: -0.52, 95% CI -0.75 to -0.30, I²=46%) | ⨁⨁◯◯ Low | CRITICAL |
| Figure 3. Postpartum depressive symptoms in prevention-oriented trials | | | | | | | | | | | | |
| 7 | Randomized clinical trials | Seriousa | Seriousa | Not serious | Not serious | Publication bias strongly suspectedc | 1074 | 1231 | - | (SMD: -0.33, 95% CI -0.57 to -0.09, I²=86%) | ⨁◯◯◯ Very Low | CRITICAL |
| Figure 4. Antenatal anxiety symptoms in prevention-oriented trials | | | | | | | | | | | | |
| 3 | Randomized clinical trials | Seriousa | Not serious | Not serious | Seriousa | Publication bias strongly suspectedc | 96 | 95 | - | (SMD:-0.03, 95% CI -0.31 to 0.25, I²=0%). | ⨁◯◯◯  Very Low | CRITICAL |
| Figure 5. Postpartum depressive symptoms in symptomatic/treatment-oriented trials | | | | | | | | | | | | |
| 3 | Randomized clinical trials | Very seriousa | Not serious | Not serious | Seriousa | Publication bias strongly suspectedc | 97 | 95 | - | (SMD: -0.38, 95% CI -0.72 to -0.04, I²=28%) | ⨁◯◯◯ Very Low | CRITICAL |
| Supplementary Figure S1. Structured physical activity on antenatal depressive symptoms in prevention-oriented trials | | | | | | | | | | | | |
| 5 | Randomized clinical trials | Seriousa | Not serious | Not serious | Not serious | Publication bias strongly suspectedc | 309 | 279 | - | (SMD: -0.59, 95% CI -0.84 to -0.34, I²=54%) | ⨁⨁◯◯ Low | CRITICAL |
| Supplementary Figure S2. Structured physical activity on postpartum depressive symptoms in prevention-oriented trials | | | | | | | | | | | | |
| 6 | Randomized clinical trials | Seriousa | Seriousb | Not serious | Seriousa | Publication bias strongly suspectedc | 935 | 1099 | - | (SMD: -0.34, 95% CI -0.62 to -0.06, I²=88%) | ⨁◯◯◯ Very Low | CRITICAL |
| Supplementary Figure S3. Physical activity performed three times per week for approximately 60 minutes on antenatal depressive symptoms in prevention-oriented trials | | | | | | | | | | | | |
| 5 | Randomized clinical trials | Seriousa | Not serious | Not serious | Not serious | Publication bias strongly suspectedc | 287 | 258 | - | (SMD: -0.62, 95% CI -0.84 to -0.40, I²=37%) | ⨁⨁◯◯  Low | CRITICAL |
| Supplementary Figure S4. Physical activity performed three times per week for approximately 60 minutes on postpartum depressive symptoms in prevention-oriented trials | | | | | | | | | | | | |
| 3 | Randomized clinical trials | Seriousa | Seriousb | Not serious | Seriousa | Publication bias strongly suspectedc | 456 | 650 | - | (SMD: -0.57, 95% CI -1.05 to -0.09, I²=92%) | ⨁◯◯◯ Very Low | CRITICAL |
| Supplementary Figure S5. Moderate-intensity physical activity on postpartum depressive symptoms in prevention-oriented trials | | | | | | | | | | | | |
| 3 | Randomized clinical trials | Seriousa | Very seriousb | Not serious | Seriousa | Publication bias strongly suspectedc | 396 | 583 | - | (MD: -1.61, CI 95% [-3.16 to -0.07]; I²=93%) | ⨁◯◯◯ Very Low | CRITICAL |

**CI:** Confidence interval; **MD:** Mean difference; **SMD:** Standardised mean difference.

Note: a. risk of bias: one level if the mean score of the studies involved in the comparison was between 6 to 8 according to the PEDro scale. Two levels if this score was less than 6 (CASHIN; MCAULEY, 2020); b. Inconsistency: heterogeneity appraised contextually (direction/magnitude of effects, CI overlap, and clinical/methodological diversity); c. Publication bias: downgraded when small-study effects or asymmetry were suspected based on the limited number of studies and visual appraisal; formal tests were not applied when fewer than 10 trials were available for an outcome. I² considered descriptively (no rigid thresholds).
