# Supplementary material for: Isolated physical activity programs during pregnancy and postpartum: Meta-analysis of randomized trials with usual-care controls on depressive and anxiety symptoms
Source: Arch Womens Ment Health. 2026 Sep 14;29(5):116. doi: 10.1007/s00737-026-01752-9 (PMC13572343; doi:10.1007/s00737-026-01752-9)
Supplement: Supplementary file 3 [file 737_2026_1752_MOESM3_ESM.docx]

***Supplement 3*.** Summary of findings and certainty of evidence using GRADE

| **Outcome and synthesis** | **Number of participants (RCTs)** | **Effect size Absolute (95% CI)** | **Certainty of the evidence**  **(GRADE)^a^** | **Comments** |
| --- | --- | --- | --- | --- |
| Figure 2. Antenatal depressive symptoms — prevention-oriented trials | A. Intervention n= 350  B. Control n= 321 | (SMD: -0.52, 95% CI: -0.75 to -0.30, I²=46%)^5,6,7^ | ⨁⨁◯◯ Low 1^,2,3,4^ | The certainty of evidence for the effect of physical activity on antenatal depressive symptoms in prevention-oriented trials was low. The pooled estimate favored physical activity, but confidence in the estimate was limited by serious risk of bias and suspected publication bias; therefore, the true effect may differ from the estimated effect. |
| Figure 3. Postpartum depressive symptoms — prevention-oriented trials | A. Intervention n= 1074  B. Control n= 1231 | (SMD: -0.33, 95% CI: -0.57 to -0.09, I²=86%)^5,6,7^ | ⨁◯◯◯ Very Low ^1,2,3,4^ | The certainty of evidence for the effect of physical activity on postpartum depressive symptoms in prevention-oriented trials was very low. Although the pooled estimate favored physical activity, confidence in the estimate was limited by serious risk of bias, substantial inconsistency, and suspected publication bias; therefore, the true effect may be markedly different from the estimated effect. |
| Figure 4. Antenatal anxiety symptoms — prevention-oriented trials | A.Intervention n= 96  B. Control n= 95 | (SMD: -0.03, 95% CI: -0.31 to 0.25, I²=0%)^5,6,7^ | ⨁◯◯◯ Very Low ^1,2,3,4^ | The certainty of evidence for the effect of physical activity on antenatal anxiety symptoms in prevention-oriented trials was very low. The pooled estimate did not show a clear effect, and confidence in the estimate was limited by serious risk of bias, imprecision, and suspected publication bias; therefore, the true effect may differ from the estimated effect. |
| Figure 5. Postpartum depressive symptoms — symptomatic/treatment-oriented trials | A. Intervention n= 97  B. Control n= 95 | (SMD: -0.38, 95% CI: -0.72 to -0.04, I²=28%)^5,6,7^ | ⨁◯◯◯ Very Low ^1,2,3,4^ | The certainty of evidence for the effect of physical activity on postpartum depressive symptoms in symptomatic/treatment-oriented trials was very low. Although the pooled estimate favored physical activity, confidence in the estimate was limited by very serious risk of bias, imprecision, and suspected publication bias; therefore, the true effect may be substantially different from the estimated effect.. |
| Supplementary Figure S1. Structured physical activity on antenatal depressive symptoms — prevention-oriented trials | A. Intervention n= 309  B. Control n= 279 | (SMD: -0.59, 95% CI: -0.84 to -0.34, I²=54%)^5,6,7^ | ⨁⨁◯◯ Low ^1,2,3,4^ | The certainty of evidence for the effect of structured physical activity on antenatal depressive symptoms in prevention-oriented trials was low. The pooled estimate favored structured physical activity, but confidence in the estimate was limited by serious risk of bias and suspected publication bias; therefore, the true effect may differ from the estimated effect. |
| Supplementary Figure S2. Structured physical activity on postpartum depressive symptoms — prevention-oriented trials | A. Intervention n= 935  B. Control n= 1099 | (SMD: -0.34, 95% CI: -0.62 to -0.06, I²=88%)^5,6,7^ | ⨁◯◯◯ Very Low ^1,2,3,4^ | The certainty of evidence for the effect of structured physical activity on postpartum depressive symptoms in prevention-oriented trials was very low. Although the pooled estimate favored structured physical activity, confidence in the estimate was limited by serious risk of bias, substantial inconsistency, imprecision, and suspected publication bias; therefore, the true effect may be markedly different from the estimated effect. |
| Supplementary Figure S3. Physical activity performed three times per week for approximately 60 minutes on antenatal depressive symptoms — prevention-oriented trials | A. Intervention n= 287  B. Control n= 258 | (SMD: -0.62, 95% CI: -0.84 to -0.40, I²=37%)^5,6,7^ | ⨁⨁◯◯ Low ^1,2,3,4^ | The certainty of evidence for the effect of physical activity performed three times per week for approximately 60 minutes on antenatal depressive symptoms in prevention-oriented trials was low. The pooled estimate favored physical activity, but confidence in the estimate was limited by serious risk of bias and suspected publication bias; therefore, the true effect may differ from the estimated effect. |
| Supplementary Figure S4. Physical activity performed three times per week for approximately 60 minutes on postpartum depressive symptoms — prevention-oriented trials | A. Intervention n= 456  B. Control n= 650 | (SMD: -0.57, 95% CI: -1.05 to -0.09, I²=92%)^7,8^ | ⨁◯◯◯ Very Low ^1,2,3,4^ | The certainty of evidence for the effect of physical activity performed three times per week for approximately 60 minutes on postpartum depressive symptoms in prevention-oriented trials was very low. Although the pooled estimate favored physical activity, confidence in the estimate was limited by serious risk of bias, substantial inconsistency, imprecision, and suspected publication bias; therefore, the true effect may be markedly different from the estimated effect. |
| Supplementary Figure S5. Moderate-intensity physical activity on postpartum depressive symptoms — prevention-oriented trials | A.Intervention  n= 396  B. Control  n= 583 | (MD: -1.61,  95% CI: -3.16  to -0.07;  I² = 93%) ^5,6,7,8^ | ⨁◯◯◯ Very Low ^1,2,3,4^ | The certainty of evidence for the effect of moderate-intensity physical activity on postpartum depressive symptoms in prevention-oriented trials was very low. Although the pooled estimate favored moderate-intensity physical activity on the EPDS scale, confidence in the estimate was limited by serious risk of bias, very serious inconsistency, imprecision, and suspected publication bias; therefore, the true effect may be markedly different from the estimated effect. |

a GRADE Working Group certainty ratings: Very low certainty indicates that the true effect is likely to be substantially different from the estimated effect. Low certainty indicates that further research is very likely to have an important impact on confidence in the estimate and is likely to change the estimate. Moderate certainty indicates that further research is likely to have an important impact on confidence and may change the estimate. High certainty indicates that further research is very unlikely to change confidence in the estimate; the true effect is likely to be close to the estimate.

b Risk of bias was downgraded one level when the mean PEDro score across studies in the comparison was 6–8 and two levels when the mean PEDro score was <6.

c Publication bias was downgraded when small-study effects or asymmetry were suspected based on the limited number of studies and visual appraisal; formal tests were not applied when fewer than 10 trials were available for an outcome.

1 Concerns with risk of bias due to lack of allocation concealment.

2 Concerns with risk of bias due to lack of blinding of participants and/or personnel.

3 Concerns with risk of bias due to unclear, absent, or insufficiently reported blinding of outcome assessors.

4 Concerns with risk of bias due to limitations in random allocation or insufficient reporting of randomization procedures.

5 Serious inconsistency based on contextual appraisal of heterogeneity, considering the direction and magnitude of effects, overlap of confidence intervals, and clinical/methodological diversity across trials.

6 Serious imprecision due to limited information size and/or confidence intervals compatible with both clinically unimportant and important effects.

7 Suspected publication bias or small-study effects based on the limited number of studies and visual appraisal.

8 Very serious inconsistency based on substantial statistical heterogeneity and clinical/methodological diversity across trials.
