# Supplementary material for: Isolated physical activity programs during pregnancy and postpartum: Meta-analysis of randomized trials with usual-care controls on depressive and anxiety symptoms
Source: Arch Womens Ment Health. 2026 Sep 14;29(5):116. doi: 10.1007/s00737-026-01752-9 (PMC13572343; doi:10.1007/s00737-026-01752-9)
Supplement: Supplementary file 4 [file 737_2026_1752_MOESM4_ESM.docx]

**Supplement 4 - Scope comparison with prior systematic reviews on physical activity and perinatal depression**

This table positions the present review against prior syntheses regarding intervention scope, comparator, temporal separation (antenatal vs postpartum), handling of baseline severity, and implementation detail.

| **Review (first author, year)** | **Isolated PA only?** | **Comparator (usual care only?)** | **Antenatal vs postpartum (separated?)** | **Baseline severity (prevention vs treatment)** | **Implementation details (modality/supervision/adherence)** | **Notes** |
| --- | --- | --- | --- | --- | --- | --- |
| **Present review** | Yes | Yes (usual care; no additional intervention) | Yes (separate analyses) | Yes (prevention-focused pooling; symptomatic trials narrated) | Detailed (modality, supervision, frequency/intensity, adherence) | Primary pooling restricted to prevention; two symptomatic/treated RCTs not pooled. |
| **Davenport 2018 (BJSM)** | Exercise-based (not strictly isolated across all trials) | Mixed (includes active comparators) | Often combined prenatal with postpartum outcomes | Not consistently stratified | Partial | Prenatal exercise with outcomes assessed prenatal/postnatal; broader inclusion than current review. |
| **Carter 2019 (Arch Womens Ment Health)** | Exercise-based (may include adjuncts) | Mixed/standard care | Postpartum focus | Mixed (prevention and treatment not consistently separated) | Partial | Targets postpartum depression; broader comparator scope than current review. |
| **Liu 2022 (Front Psychol)** | Yes (exercise as the only intervention) | Mixed (inactive + active) | Perinatal mixed | No formal prevention vs treatment stratification | Partial | Broad perinatal scope with heterogeneous comparators and interventions. |
| **He 2023 (J Affect Disord)** | Physical activity interventions (varied) | Mixed | Perinatal mixed | Not consistent | Partial | Wider net of PA interventions; comparator types vary. |
| **Ji 2024 (J Affect Disord)** | Yes | Usual care / no exercise only (not mixed) | Perinatal (prevention and treatment) | Mixed | Limited–Partial | Focus on modality effects across prevention/treatment contexts. |
| **Shuai 2024 (BMC Public Health; NMA)** | Multiple PA interventions | Mixed (network of comparators) | Perinatal mixed | Not consistently separated | Limited–Partial | Network meta-analysis across varied interventions and comparators. |

Abbreviations: PA, physical activity; NMA, network meta-analysis. “Isolated PA” indicates trials where physical activity was the only intervention component. “Usual care only” excludes co-interventions beyond routine care. Classifications are based on each review’s eligibility criteria, comparator definitions, and synthesis choices.
