# Supplementary material for: Isolated physical activity programs during pregnancy and postpartum: Meta-analysis of randomized trials with usual-care controls on depressive and anxiety symptoms
Source: Arch Womens Ment Health. 2026 Sep 14;29(5):116. doi: 10.1007/s00737-026-01752-9 (PMC13572343; doi:10.1007/s00737-026-01752-9)
Supplement: Supplementary file 5 [file 737_2026_1752_MOESM5_ESM.docx]

***Supplementary Figures***

***Supplementary Figure S1.*** *Impact of structured physical activity on antenatal depressive symptoms compared with no intervention in prevention-oriented trials.*

***Supplementary Figure S2.*** *Impact of structured physical activity on postpartum depressive symptoms compared with no intervention in prevention-oriented trials.*

***Supplementary Figure S3.*** *Impact of physical activity performed three times per week for approximately 60 minutes on antenatal depressive symptoms compared with no intervention in prevention-oriented trials.*

***Supplementary Figure S4.*** *Impact of physical activity performed three times per week for approximately 60 minutes on postpartum depressive symptoms compared with no intervention in prevention-oriented trials.*

***Supplementary Figure S5****. Impact of moderate-intensity physical activity on postpartum depressive symptoms compared with no intervention in prevention-oriented trials (MD on EPDS, 0-30).*
